# Surface Modification by Polyzwitterions of the Sulfabetaine-Type, and Their Resistance to Biofouling

**DOI:** 10.3390/polym11061014

**Published:** 2019-06-08

**Authors:** Eric Schönemann, André Laschewsky, Erik Wischerhoff, Julian Koc, Axel Rosenhahn

**Affiliations:** 1 Department of Chemistry, University Potsdam, Karl-Liebknecht-Str. 24-25, 14476 Potsdam-Golm, Germany; eschoenemann@uni-potsdam.de; 2 Fraunhofer Institute of Applied Polymer Research IAP, 14476 Potsdam-Golm, Germany; erik.wischerhoff@iap.fraunhofer.de; 3 Analytical Chemistry – Biointerfaces, Ruhr University Bochum, 44780 Bochum, Germany; julian.koc@rub.de (J.K.); axel.rosenhahn@rub.de (A.R.)

**Keywords:** polyzwitterion, sulfabetaine, sulfobetaine, polymer thin films, photo crosslinking, C,H insertion crosslinking (CHic), protein adsorption, anti-fouling materials

## Abstract

Films of zwitterionic polymers are increasingly explored for conferring fouling resistance to materials. Yet, the structural diversity of polyzwitterions is rather limited so far, and clear structure-property relationships are missing. Therefore, we synthesized a series of new polyzwitterions combining ammonium and sulfate groups in their betaine moieties, so-called poly(sulfabetaine)s. Their chemical structures were varied systematically, the monomers carrying methacrylate, methacrylamide, or styrene moieties as polymerizable groups. High molar mass homopolymers were obtained by free radical polymerization. Although their solubilities in most solvents were very low, brine and lower fluorinated alcohols were effective solvents in most cases. A set of sulfabetaine copolymers containing about 1 mol % (based on the repeat units) of reactive benzophenone methacrylate was prepared, spin-coated onto solid substrates, and photo-cured. The resistance of these films against the nonspecific adsorption by two model proteins (bovine serum albumin—BSA, fibrinogen) was explored, and directly compared with a set of references. The various polyzwitterions reduced protein adsorption strongly compared to films of poly(*n*-butyl methacrylate) that were used as a negative control. The poly(sulfabetaine)s showed generally even somewhat higher anti-fouling activity than their poly(sulfobetaine) analogues, though detailed efficacies depended on the individual polymer–protein pairs. Best samples approach the excellent performance of a poly(oligo(ethylene oxide) methacrylate) reference.

## 1. Introduction

Polyzwitterions—or synonymous polybetaines—bear within their constitutional repeat unit (CRU) pairs of anionic and cationic groups. Although they are characterized by a high density of ions attached to the polymer chain and therefore, strong electrostatic intra- and interpolymer interactions may occur, the net charge of polyzwitterions is inherently zero under normal conditions. Hence, polyzwitterions represent a special subclass of polyampholytes featuring very particular properties, including high polarity (and thus often high hydrophilicity), anti-polyelectrolyte behavior and stimuli-responsiveness [1,2,3]. By virtue of this exceptional property profile, they have increasingly attracted interest due to their great potential for diverse applications, such as biocompatibilization, cryo preservation, lubrication, smart self-assembly, and low-fouling surfaces in aqueous environments [4,5,6,7,8,9,10,11,12,13]. 

**Scheme 1:**
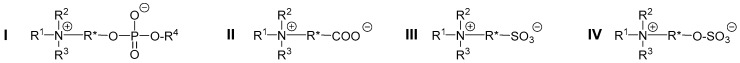
Generalized structure of major polyzwitterion classes. From left to right: Ammonio phosphates (I, specifically for R* = –CH_2_–CH_2_–: phosphatidylcholines), ammonio carboxylates (II, carboxybetaines), ammonio sulfonates (III, sulfobetaines), and ammonio sulfates (IV, sulfabetaines).

Compared to the mostly employed anti-fouling materials based on poly(ethylene oxide), polyzwitterions excel for instance in their chemical stability, in particular against oxygen and other oxidants such as chlorine [14,15,16]. Although a large variety of zwitterionic moieties has been incorporated into polymers, the vast majority of studies have focused hitherto on three poly-zwitterion families, namely poly(phosphatidylcholine)s [10,17,18,19,20], poly(carboxybetaine)s [21,22,23,24,25,26,27,28,29], and poly(sulfobetaine)s [30,31,32,33,34,35,36,37,38,39]. While the interest in the former family is due to their similarity to phospholipids [40,41,42], the latter two families are characterized by good stability, high structural variability, and straightforward synthetic accessibility [2,3]. Recently, a forth family of poly-zwitterions was proposed as a complement, named poly(sulfabetaine)s [43,44], that combines the cationic ammonium group with the anionic sulfate group in their zwitterionic constitutional repeat unit (CRU; Scheme 1). 

In an analogy to the synthesis of sulfobetaines by the ring-opening alkylation of tertiary amines with cyclic sulfonate esters, sulfabetaines can be readily synthesized without salt contaminants by the ring-opening alkylation of tertiary amines with cyclic sulfate esters. Despite the versatility of this approach, reports on poly(sulfabetaine)s have been scarce so far [43,44,45,46,47,48,49,50,51,52]. Most probably this apparent lack of interest is not due to the somewhat reduced hydrolytic stability of sulfabetaines compared to sulfobetaine analogues (which is still reasonably high [48,50]), but mostly due to the very low solubility of poly(sulfabetaine)s in water. In fact, the poly(sulfabetaine)s described so far exhibit typically upper critical solution temperatures (UCSTs) above 100 °C in pure water [43,47,49,50]. Large amounts of low molar mass salts must be added to induce their solubility at ambient temperature (“salting-in”). Although in special situations, e.g., in the case of thermo-responsive materials under physiologically relevant situations, this may be an advantage [51], the need of a strong salting-in scenario is mostly considered to be problematic.

**Figure 1:**
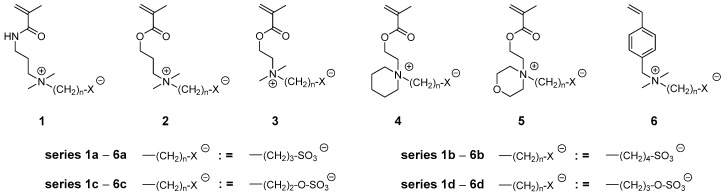
Examples of zwitterionic methacrylic and styrenic sulfobetaine (series a–b) and sulfabetaine (series c–d) monomers studied: Series a = 3-ammonio propanesulfonates, series b = 4-ammonio butanesulfonates, series c = 2-ammonio ethylsulfates, and series d = 3-ammonio propylsulfates.

Recently, we undertook an extended study on the structural variation of poly(sulfobetaine methacrylate)s (as exemplified in Figure 1) and its effect on the phase behavior in aqueous solution, in particular on the position of the aqueous UCST transition [38].

While empirically, the transition temperature could be effectively adjusted between 0 and 100 °C by straightforward variations of the chemical structure of the CRU, the results could not be rationalized by simple rules that enable a reliable prediction of the phase behavior of new variants for such stimuli-responsive polymers. The studies nevertheless demonstrated the strong effects of apparently small chemical variations on their water-solubility. In particular polymers of monomers 2a (P−2a), 4a (P−4a), and 5a (P−5a) were found to feature unusually low UCST transitions, and thus to exhibit only very small miscibility gaps in water, if at all [38]. Based on these findings, we synthesized two series of poly(sulfabetaine)s (Figure 1, series c and d) that are analogously designed to previously described series of poly(sulfobetaine)s (Figure 1, series a and b), and explored them with respect to their water-solubility and possible improvements. Furthermore for selected examples, we explored their utility for low-fouling coatings, as initial studies on polymers P−1d and P−3d had indicated good resistance against the adsorption of proteins [12,52].

## 2. Materials and Methods

### 2.1. Materials

Initiator 2,2′-azobisisobutyronitrile “AIBN” (Sigma Aldrich Chemie GmbH, Taufkirchen, Germany, 98%) was crystallized from methanol prior to use. Poly(ethylene glycol) methyl ether methacrylate (“OEGMA”, *M*_r_ = 500, Sigma Aldrich, containing 100 ppm of monomethylhydroquinone “MEHQ” and 200 ppm of 2,6-di-tert-butyl-4-methylphenol “BHT” as inhibitors) was purified prior to use by filtration through a bed of aluminum oxide (“ALOX” 90 active neutral, 70–230 mesh ASTM 0.063–0.2 mm, Merck, Darmstadt, Germany). Butyl methacrylate (“BMA”, Sigma Aldrich, containing 10 ppm of monomethylhydroquinone “MEHQ” as an inhibitor) was purified prior to use by distillation. Monomers 3-[*N*-3-(methacrylamido)propyl-*N*,*N*-dimethyl]ammonio propane-1-sulfonate 1a (“SPP”, gift from Raschig GmbH, Ludwigshafen, Germany) and 3-[*N*-2-(methacryloyloxy)ethyl-*N,N*-dimethyl]ammonio propane-1-sulfonate 3a (“SPE”, Sigma Aldrich, ≥97%) were used as received. The synthesis of sulfabetaine monomers 1c, 1d, 3c, 3d, 6c, and 6d is described elsewhere [50], as is the synthesis of the tertiary amine functionalized methacrylate intermediates 3-(dimethyl-amino)propyl methacrylate, 2-(piperidin-1-yl)ethyl methacrylate, and 2-morpholino-1-yl-ethyl methacrylate [38]. Photocrosslinker 2-(4-benzoylphenoxy)ethyl methacrylate (BPEMA) was synthesized as described before [53].

Reagents and solvents 1,3,2-dioxathiolane 2,2-dioxide (“ethylenesulfate”, TCI Deutschland GmbH, Eschborn, Germany, ≥98.0%), 1,3,2-dioxathian 2,2-dioxide (“propylenesulfate”, TCI ≥ 98.0%), acetonitrile (Carl Roth GmbH, Karlsruhe, Germany, ≥99,9%), acetone (VWR International GmbH, Darmstadt, Germany, 100.0%), trifluoroethanol (Roth, 99.8%), methanol (VWR, ≥98.5%), ethanol (Chemsolute/Th. Geyer, Renningen, Germany, ≥99.5%), sodium chloride (Chemsolute, ≥99.0%), deuterium oxide (VWR, ≥99.90 atom% deuterium), sodium carbonate (Acros Organics/Fisher Scientific GmbH, Schwerte, Germany, purum), phosphate buffered saline tablets (“PBS”, Sigma-Aldrich, Lot BCBF6911), 3-(trimethylsilyl) propionic acid-d4 sodium salt (Acros, 98 atom% deuterium), and 1,1,1,3,3,3-hexafluoro-2-propanol “HFIP” (99%, Fluorochem, Hadfield, UK) were used as received if not stated otherwise. Albumin from bovine serum Alexa fluor^TM^ 647 conjugate (catalogue no. A34785, Lot: 1774711) and fibrinogen from human plasma Alexa fluor^TM^ 647 conjugate (catalogue no. F35200, Lot: 1780253) were supplied by Thermo Fisher Scientific GmbH, Berlin, Germany and used as received. Water was deionized and further purified by a Millipore Milli-Q Plus water purification system (Merck Millipore, Darmstadt, Germany), resistivity 18 mΩ·cm^−1^.

### 2.2. Synthetic Methods and Procedures 

#### 2.2.1. Synthesis of Sulfabetaine Monomers

2-((3-(methacryloyloxy)propyl)dimethylammonio) ethyl-1-sulfate (2c): 3-(dimethylamino)-propyl methacrylate (6.06 g, 35.3 mmol, 1.10 eq.) was added to a solution of ethylenesulfate (4.00 g, 32.2 mmol, 1.00 eq.) in dry acetonitrile (24 mL). After adding three drops of nitrobenzene, the mixture was stirred for 40 h at 50 °C. In the course of the reaction, a colorless precipitate of the desired product was formed. Then, the mixture was cooled to ambient temperature, 50 mL of acetonitrile was added, and the mixture was precipitated into a large excess of diethyl ether; the solid precipitate was filtered off and redispersed in acetonitrile. The procedure was repeated once more, the isolated crude product washed thrice with a few mL of acetone, and crystallized from acetonitrile/methanol (15:1, *v*/*v*). The pure monomer was obtained as colorless powder (7.80 g, 82% yield).

^1^H NMR (300 MHz, D_2_O) δ 6.21–6.16 (m, 1H, a‘), 5.80–5.74 (m, 1H, a‘‘), 4.59–4.48 (m, 2H, j), 4.33 (t, *J* = 5.9 Hz, 2H, e), 3.86–3.77 (m, 2H, i), 3.66–3.54 (m, 2H, g), 3.25 (s, 6H, h), 2.37–2.22 (m, 2H, g), 1.99–1.95 (m, 3H, c).

^13^C NMR (75 MHz, D_2_O) δ = 169.64 (d), 136.03 (b), 127.28 (a), 63.02 (2C, i + g), 62.13 (e), 61.90 (j), 51.91 (2C, h), 22.14 (f), 17.56 (c).

High resolution mass spectra (HR-MS; ESI). Calculated: 296.1168 [M+H]^+^, found: 296.1176. 

Elemental analysis (C_11_H_21_NO_6_S). Calculated: C = 44.73%, H = 7.17%, N = 4.74%, S = 10.85%, O = 32.50%; found: C = 44.06%, H = 7.24%, N = 4.73%, S = 11.02%.

FT-IR (selected bands cm^−1^): 3046, 3012, 2962, 2902, 1716, 1634, 1488, 1464, 1324, 1302, 1245, 1220, 1177, 1064, 1041.

2-(1-(2-(methacryloyloxy)ethyl)piperidinio-1-yl)ethyl-1-sulfate (4c): Analogously to monomer 2c, monomer 4c was synthesized, purified, and isolated analogously as colorless powder in 74% yield, starting from 2-(piperidin-1-yl)ethyl methacrylate. 

^1^H NMR (300 MHz, D_2_O) δ = 6.21–6.14 (m, 1H, a‘), 5.81 (s, 1H, a‘‘), 4.72–4.62 (m, 2H, e), 4.58–4.49 (m, 2H, k), 4.03–3.89 (m, 4H, j + f), 3.66–3.55 (m, 4H, g + g‘), 2.06–1.93 (m, 7H, h + h‘ + c), 1.81–1.69 (m, 2H, i).

^13^C NMR (75 MHz, D_2_O) δ = 168.92 (d), 135.47 (b), 127.93 (a), 61.38 (3C, k + j + f), 58.39 (e), 58.23 (2C, g + g’), 20.60 (i), 19.63 (2C, h + h’), 17.49 (c).

HR-MS (ESI). Calculated: 322.1324 [M+H]^+^; found: 322.1325. 

Elemental analysis (C_13_H_23_NO_6_S). Calculated: C = 48.58%, H = 7.21%, N = 4.36%, S = 9.98%, O = 29.87%; found: C = 48.52%, H = 7.25%, N = 4.34%, S = 10.18%.

FT-IR (selected bands cm^−1^): 3008, 2955, 2867, 1711, 1628, 1459, 1318, 1265, 1237, 1158, and 1031.

2-(4-(2-(methacryloyloxy)ethyl)morpholinio-4-yl)ethyl-1-sulfate (5c): Analogously to monomer 2c, monomer 5c was synthesized, purified, and isolated as colorless powder in 85% yield, starting from 2-morpholinoethyl methacrylate. 

^1^H NMR (300 MHz, D_2_O) δ = 6.27–6.14 (m, 1H, a‘), 5.92–5.77 (m, 1H, a‘‘), 4.78–4.70 (m, 2H, j), 4.65–4.51 (m, 2H, e), 4.25–4.08 (m, 8H, f + h + h‘ + i), 3.86–3.75 (m, 4H, g + g‘), 2.02–1.97 (m, 3H, c).

^13^C NMR (75 MHz, D_2_O) δ = 168.53 (d), 135.43 (b), 128.07 (a), 61.32 (e), 60.51 (2C, g + i), 59.89 (2C, h + h’), 58.79 (2C, g’ + f), 58.13 (j), 17.54 (c). 

HR-MS (ESI). Calculated: 324.1122 [M+H]^+^; found: 324.1117. 

Elemental analysis (C_12_H_21_NO_7_S). Calculated: C = 44.57%, H = 6.55%, N = 4.33%, S = 9.91%, O = 34.63%; found: C = 43.90%, H = 6.68%, N = 4.42%, S = 10.03%.

FT-IR (selected bands in cm^−1^): 3012, 2960, 2891, 1709, 1629, 1442, 1320, 1232, 1160, 1131, and 1027.

3-((3-(methacryloyloxy)propyl)dimethylammonio)propyl-1-sulfate (2d): 3-(dimethylamino)-propyl methacrylate (6.82 g, 39.9 mmol, 1.10 eq.) was added to a solution of propylenesulfate (5.00 g, 36.2 mmol, 1.00 eq.) in dry acetonitrile (27 mL). After adding three drops of nitrobenzene, the mixture was stirred for 40 h at 50 °C. In the course of the reaction, a colorless precipitate of the desired product was formed. Then, the mixture was cooled to ambient temperature, 50 mL of acetonitrile was added, and the mixture was precipitated into a large excess of diethyl ether; the solid precipitate was filtered off and redispersed in acetonitrile. The procedure was repeated once more, the isolated crude product washed thrice with a few mL of acetone, and crystallized from acetonitrile/methanol (15:1, *v*/*v*). The pure monomer was obtained as colorless powder (9.83 g, 88% yield).

^1^H NMR (300 MHz, D_2_O) δ = 6.25–6.15 (m, 1H, a‘), 5.85–5.74 (m, 1H, a‘‘), 4.33 (t, *J* = 5.8 Hz, 2H, e), 4.20 (t, *J* = 5.7 Hz, 2H, k), 3.63–3.47 (m, 4H, g + i), 3.18 (s, 6H, h), 2.37–2.20 (m, 4H, f + j), 2.03–1.94 (m, 3H, c).

^13^C NMR (75 MHz, D_2_O) δ = 169.60 (d), 135.97 (b), 127.24 (a), 65.46 (k), 62.01 (e), 61.63 (g), 61.31 (i), 51.06 (h), 22.61 (f), 21.96 (j), 17.52 (c).

HR-MS (ESI). Calculated: 310.1324 [M+H]^+^; found: 310.1336. 

Elemental analysis (C_12_H_23_NO6S). Calculated: C = 46.59%, H = 7.49%, N = 4.53%, S = 10.36%, O = 31.03%; found: C = 49.85%, H = 7.70%, N = 4.20%, S = 9.57%.

FT-IR (selected bands cm^−1^): 2954, 2887, 1709, 1631, 1485, 1452, 1322, 1225, 1170, 1045, 1028.

3-(1-(2-(methacryloyloxy)ethyl)piperidinio-1-yl)propyl-1-sulfate (4d): Analogously to monomer 2d, monomer 4d was synthesized, purified, and isolated analogously as colorless powder in 87% yield, starting from 2-(piperidin-1-yl)ethyl methacrylate. 

^1^H NMR (300 MHz, D_2_O) δ 6.14–6.02 (m, 1H, a‘), 5.78–5.66 (m, 1H, a‘‘), 4.59–4.50 (m, 2H, e), 4.09 (t, *J* = 5.6 Hz, 2H, l), 3.81–3.70 (m, 2H, f), 3.57–3.47 (m, 2H, j), 3.48–3.35 (m, 4H, g + g‘), 2.21–2.04 (m, 2H, k), 1.92–1.79 (m, 7H, c + h + h‘), 1.75–1.55 (m, 2H, i).

^13^C NMR (75 MHz, D_2_O) δ = 168.74 (d), 135.47 (b), 128.00 (a), 65.62 (l), 60.67 (2C, g + g’), 58.12 (e), 57.12 (j), 56.84 (f), 21.72 (k), 20.71 (i), 19.54 (2C, h + h’), 17.50 (c).

HR-MS (ESI). Calculated: 336.1460 [M+H]^+^; found: 336.1460. 

Elemental analysis (C_14_H_25_NO_6_S). Calculated: C = 50.13%, H = 7.51%, N = 4.18%, S = 9.56%, O = 28.62%; found: C = 49.84%, H = 7.75%, N = 4.18%, S = 9.60%.

FT-IR (selected bands in cm^−1^): 2954, 2887, 1709, 1631, 1485, 1452, 1322, 1225, 1170, and 1045.

3-(4-(2-(methacryloyloxy)ethyl)morpholinio-4-yl)propyl-1-sulfate (5d): Analogously to monomer 2d, monomer 5d was synthesized, purified, and isolated analogously as colorless powder in 50% yield, starting from 2-morpholinoethyl methacrylate. 

^1^H NMR (300 MHz, D_2_O) δ = 6.22–6.14 (m, 1H), 5.88–5.77 (m, 1H), 4.75–4.66 (m, 2H, e), 4.24–4.18 (m, 2H, k), 4.18–4.11 (m, 4H, h + h’), 4.07–3.98 (m, 2H, f), 3.87–3.77 (m, 2H, i), 3.77–3.67 (m, 4H, g + g’), 2.36–2.19 (m, 2H, j), 2.01–1.93 (m, 3H).

^13^C NMR (75 MHz, D_2_O) δ = 168.57 (d), 135.34 (b), 128.07 (a), 65.38 (k), 60.38 (2C, h + h’), 59.17 (2C, g + i), 57.97 (g’), 57.60 (e), 57.19 (f), 21.64 (j), 17.49 (c). 

HR-MS (ESI). Calculated: 338.1273 [M+H]^+^; found: 338.1273. 

Elemental analysis (C_13_H_23_NO_7_S). Calculated: C = 46.28%, H = 6.87%, N = 4.15%, S = 9.50%, O = 33.19%; found: C = 44.93%, H = 6.86%, N = 4.29%, S = 9.77%.

FT-IR (selected bands in cm^-1^): 3023, 2998, 2966, 2893, 1718, 1636, 1488, 1453, 1324, 1247, 1220, 1160, 1110, 1034.

The NMR spectra of the monomers are displayed in Appendix A, Figure A1, Figure A2, Figure A3, Figure A4, Figure A5, Figure A6, Figure A7, Figure A8, Figure A9, Figure A10, Figure A11 and Figure A12.

#### 2.2.2. Polymer Synthesis

The synthesis of sulfabetaine homopolymers P−1d, P−3c, P−3d, P−6c, and P−6d was reported before [50]. The synthesis of photo-crosslinkable copolymer P−x1d containing ca. 1 mol % of benzophenone monomer BPEMA as well of the photo-crosslinkable references P−x1a, P−x3a, P−xOEGMA, and P−xBMA was described elsewhere [52]. The other sulfabetaine homopolymers and copolymers with ca. 1 mol % of photocrosslinker BPEMA of the various monomers of series 1c–6c and 2d−6d are synthesized analogously, via solution polymerization in dry trifluoroethanol (TFE) employing initiator azobisisobutyronitrile (AIBN, 0.5 mol % relative to monomer). After dilution by ultrapure water, the mixture was dialyzed against ultrapure water, using a membrane type ZelluTrans (Carl Roth GmbH, Karlsruhe, Germany) with a nominal molecular weight cut off “MWCO” of 3500 g mol^−1^, and lyophilized. The sulfabetaine polymers are typically obtained in high yields (>75%) as hygroscopic and colorless solids. The details of polymer preparation are compiled in Table 1.

Basic analytical data of the new homopolymers:

P−x2c:
  ^1^H NMR (300 MHz, D_2_O (saturated with NaCl)) δ = 5.1–4.5 (br, 4H, k + e), 4.4–4.1 (br, 2H, i), 4.1–3.9 (br, 2H, g), 3.9–3.5 (br, 6H, h), 3.1–1.6 (br, 7H, f + a), 1.9–0.6 (br, 3H, c).
  Elemental analysis (C_11_H_21_NO_6_S, *M*_CRU_ = 295.11 g∙mol^−1^): Calculated: C = 44.73%, H = 7.17%, N = 4.74%, S = 10.85%, O = 32.50%; found: C = 42.81%, H = 7.61%, N = 4.49%, S = 10.65%.
  FT-IR (selected bands, cm^−1^): 2972, 1727, 1965, 1491, 1263, 1232, 1160, 1068, and 1035.
P−x2d:
  ^1^H NMR (300 MHz, D_2_O (saturated with NaCl)) δ = 4.7–4.4 (br, 4H, e + k), 4.1–3.8 (br, 4H, g + i), 3.7–3.5 (br, 6H, h), 2.9–2.0 (br, 6H, f + j + a), 1.8–1.0 (br, 3H, c).
  Elemental analysis (C_12_H_23_NO_6_S, *M*_CRU_ = 309.12 g∙mol^−1^): Calculated: C = 46.59%, H = 7.49%, N = 4.53%, S = 10.36%, O = 31.03%; found: C = 42.33%, H = 8.06%, N = 4.11%, S = 9.76%.
  FT-IR (selected bands, cm^−1^): 2967, 2894, 1721, 1640, 1484, 1220, 1161, 1065, and 1027.
P−x4c:
  ^1^H NMR (300 MHz, D_2_O (saturated with NaCl)) δ = 5.3–4.9 (br, 4H, k + e), 4.6–3.2 (br, 8H, g + i + h‘ + h‘‘), 3.2–0.6 (br, 11H, l‘ + l‘‘ + m + a + c).
  Elemental analysis (C_13_H_23_NO_6_S, *M*_CRU_ = 321.12 g∙mol^−1^): Calculated: C = 48.58%, H = 7.21%, N = 4.36%, S = 9.98%, O = 29.87%; found: C = 45.39%, H = 7.56%, N = 4.09%, S = 9.70%.
  FT-IR (selected bands, cm^−1^): 2959, 1729, 1647, 1484, 1263, 1229, 1149, and 1031.
P−x4d:
  ^1^H NMR (300 MHz, D_2_O (saturated with NaCl)) δ = 5.9–3.3 (br, e + g + i + k + h‘ + h‘‘), 3.3–0.2 (br, a + c + l‘ + l‘‘ + m + j).
  Elemental analysis (C_14_H_25_NO_6_S, *M*_CRU_ = 335.14 g∙mol^−1^): Calculated: C = 50.13%, H = 7.51%, N = 4.18%, S = 9.56%, O = 28.62%; found: C = 45.48%, H = 7.68%, N = 3.71%, S = 8.45%.
  FT-IR (selected bands, cm^−1^): 2954, 1725, 1643, 1463, 1222, 1176, 1149, 1061, and 1030.
P−x5c:
  Insufficient solubility in D_2_O, D_2_O saturated with NaCl, or standard deuterated solvents for taking ^1^H NMR spectra.
  Elemental analysis (C_12_H_21_NO_7_S, *M*_CRU_ = 323.10 g∙mol^−1^): Calculated: C = 44.57%, H = 6.55%, N = 4.33%, S = 9.91%, O = 34.63%; found: C = 41.90%, H = 7.04%, N = 4.09%, S = 9.62%.
  FT-IR (selected bands, cm^−1^): 2994, 2891, 1728, 1639, 1480, 1264, 1231, 1132, and 1026.
P−x5d:
  Insufficient solubility in D_2_O, D_2_O saturated with NaCl, or standard deuterated solvents for taking ^1^H NMR spectra.
  Elemental analysis (C_13_H_23_NO_7_S, *M*_CRU_ = 337.12 g∙mol^−1^): Calculated: C = 46.28%, H = 6.87%, N = 4.15%, S = 9.50%, O = 33.19%; found: C = 42.52%, H = 7.12%, N = 3.80%, S = 9.09%.
  FT-IR (selected bands, cm^−1^): 2969, 2897, 1728, 1638, 1481, 1259, 1226, 1129, 1065, 1024.

**Figure A13:**
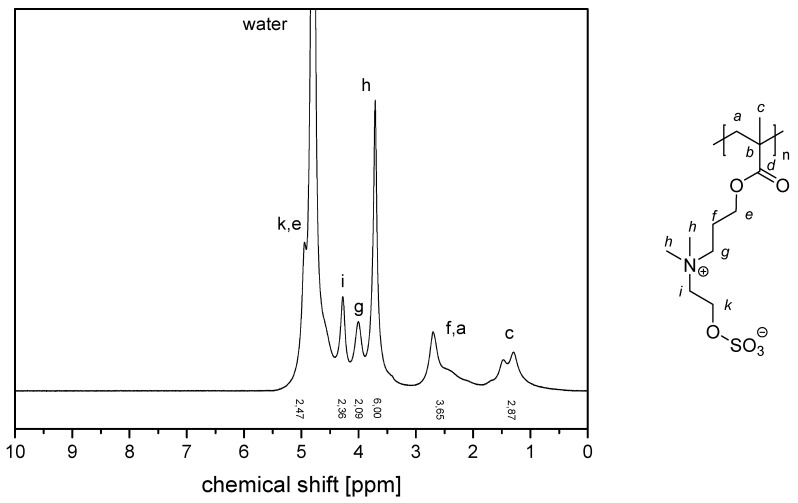
^1^H NMR-spectrum of polymer P−2c in D_2_O saturated with NaCl.

**Figure A14:**
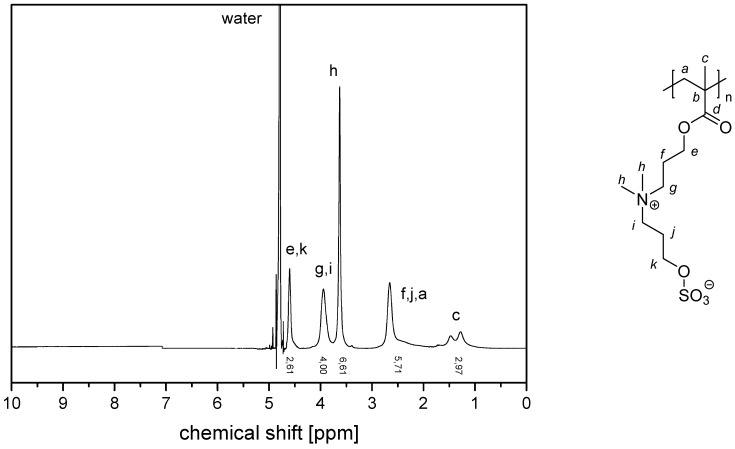
^1^H NMR-spectrum of polymer P−2d in D_2_O saturated with NaCl.

**Figure A15:**
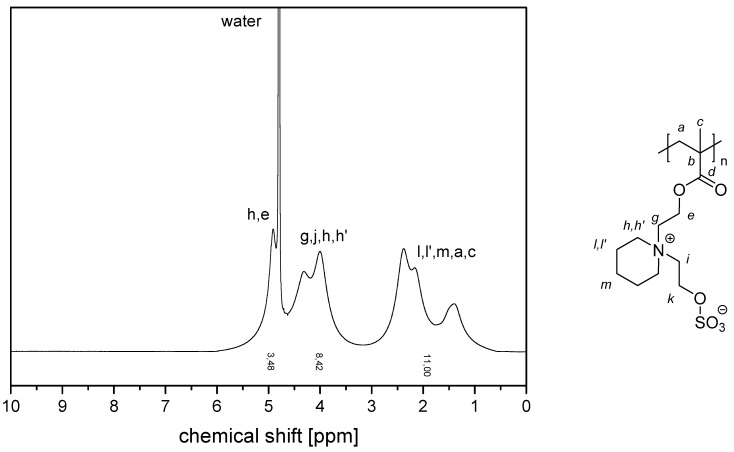
^1^H NMR-spectrum of polymer P−4c in D_2_O saturated with NaCl.

**Figure A16:**
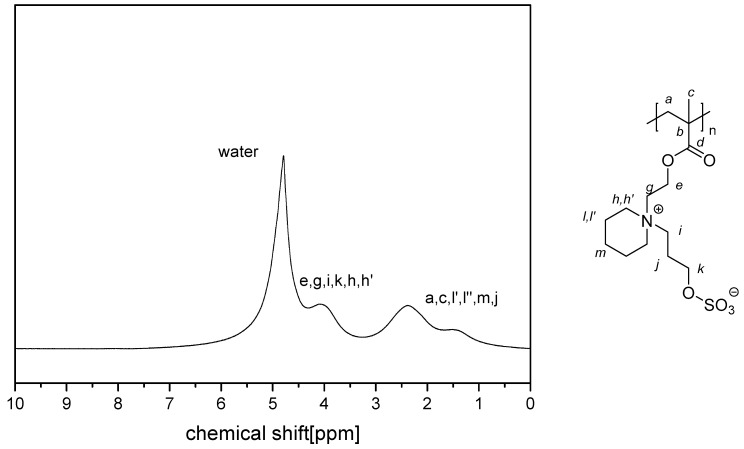
^1^H NMR-spectrum of polymer P−4d in D_2_O saturated with NaCl.

The NMR spectra of the water-soluble homopolymers are displayed in Appendix A, Figure A13, Figure A14, Figure A15 and Figure A16.

### 2.3. Instrumentation and Methods 

^1^H and ^13^C NMR spectra, ^1^H-^1^H-correlation spectra (COSY) and ^1^H-^13^C-hetero-nuclear multiple quantum coherence spectra (HMQC) were recorded with an Avance 300 spectrometer (Bruker, Billerica, MA, USA, 300 MHz and 75 MHz, respectively) at ambient temperature in deuterated solvents. ^13^C spectra were recorded in the ^1^H-broad band decoupling mode and attached proton test (ATP) mode, respectively. Solvent signals or 3-(trimethylsilyl)propionic-2,2,3,3-d_4_ acid sodium salt were used as internal shift references. High resolution mass spectra (HR-MS) were recorded with a Thermo Scientific ESI-Q-TOF micro (quadrupole-time of flight; Thermo Fisher Scientific, Waltham, MA, USA) with electrospray ionization (ESI) using water as the solvent. Element analysis was conducted by using a Vario ELII microanalyzer (Elentar Analysensysteme, Hanau, Germany). FT-IR spectra were recorded in a N_2_ purged atmosphere with a Nicolet Nexus FT-IR spectrometer (Thermo Fisher Scientific) equipped with an attenuated total reflection (ATR) smart endurance element. Absorption spectra in solution were recorded with a UV/VIS/NIR two beam spectrometer (Lamda 19, Perkin Elmer, Waltham, MA, USA). Thermogravimetric analysis (TGA) was conducted under N_2_ purged atmosphere with a TGA/SDTA 851e (Mettler-Toledo GmbH, Giessen, Germany) using a heating rate of 10 K min^−1^. The occurrence of thermal transitions was verified by differential scanning calorimetry with an apparatus DSC 822e (Mettler-Toledo), using heating and cooling rates of 10 K min^−1^. Temperature accuracy was calibrated with the onset of the melting peaks of indium (156.6 °C) and of zinc (419.6 °C). Size exclusion chromatography (SEC) for polyzwitterions was run with an apparatus SEC3010 (WGE-Dr. Bures, Dallgow-Döberitz, Germany) equipped with a refractive index detector and PL-HFIP gel columns (Agilent Technologies, Santa Clara, CA, USA), using hexafluoroisopropanol (HFIP) containing 50 mM of sodium trifluoroacetate as eluent (flow rate 0.8 mL min^−1^), and poly(methyl methacrylate) “PMMA” standards (500 to 520,000 Da, narrowly distributed, PSS Polymer Standard Service, Mainz, Germany) for calibration.

### 2.4. Film Preparation and Protein Adsorption Studies

Pieces of 2.0 cm × 2.0 cm cut from silicon wafers (0.725 mm thickness, orientation <100> front polished, Silicon Materials, Kaufering, Germany). Silicon, quartz (Suprasil, 45 mm × 12 mm × 1 mm, Hellma Analytics, Müllheim, Germany) and glass substrates (2.5 cm × 2.5 cm, cut from microscope slides, VWR) were washed and hydrophilized for 6 h in a solution of 100 mg of potassium permanganate in 40 mL of concentrated sulfuric acid. The substrates were washed three times with water, then with ethanol and finally with toluene. Subsequently, their surfaces were modified via silanization to enable the photo-grafting of the copolymers. Samples were immersed in 1 vol % solutions of (3-aminopropyl) dimethylethoxysilane in toluene, for 12 h. Then, they were taken out of the solutions, washed with toluene and ethanol, and dried under nitrogen flow. Solutions of the copolymers in TFE were spin-cast onto the pre-treated substrates with a spin-coater (KL-SCV, Schaefer technologies, Langen, Germany). Exceptionally, reference P−xBMA was spin-cast from ethyl acetate solutions for experiments using fluorescence microscopy, as films cast from TFE solutions are rather rough [52]. One droplet of a 1–2.5 wt % solution was dispensed on the rotating wafer, and the copolymer was cast at 40–80 rotations per seconds for 10 s. Precise concentrations and rotation speeds were adjusted for each sample to produce equally thin coatings of 110 ± 10 nm thickness. Films were cured for 30 min under UV-light in a UVACUBE 100 radiation chamber (Hönle, Gräfelfing, Germany) at ambient temperature in air, using a 100 W iron doped mercury vapor lamp equipped with sheet glass as a filter (spectral cut off 310 nm). The distance of the sample from the light source was 20 cm. After photo-curing, the samples were immersed in three water washing baths, while being agitated, then removed, dried by a gentle nitrogen stream, and stored at ambient temperature in the dark.

Film thicknesses were determined in the dry state by ellipsometry. An apparatus Multiscope from Optrel GbR (Kleinmachnow, Germany) was used, which was equipped with a HeNe Laser (632.8 nm wavelength) in a null ellipsometer configuration, with an angle of incidence of 70°. Dry film thicknesses on silicon supports were calculated by the software “Elli”, version 5.2 (Optrel GbR, Kleinmachnow, Germany), using a four-layer model with the following parameters: Layer 1: Air (*n* = 1.000, *k* = 0), layer 2: Organic layer (*n* = 1.4800, *k* = 0), layer 3: SiO_2_ (*d* = 1.0 nm, *n* = 1.4580, *k* = 0), layer 4: Silicon (*n* = 3.8858, *k* = −0.0200). 

Water-air contact angles were determined by the sessile drop method using an apparatus OCA 15EC equipped with the software module SCA 20 (Dataphysics Instruments, Filderstadt, Deutschland). Drops of 5 μL of Millipore water were placed on the sample, and pictures were taken with a USB-camera after 5 s. The given values were arithmetic averages of measurements of eight samples taken at two different positions. 

The adsorption of fluorophore-labeled proteins was studied with a fluorescence microscope type CKX41 (Olympus Europa GmbH, Hamburg, Germany), using the filter set Cy5 ET (AHF Analysentechnik AG, Tübingen, Deutschland). Fluorescence micrographs were taken with 5 s of exposure, using tenfold magnification and signal amplification of 7.2 db. The relative fluorescence intensities were quantified using the software cellSens Dimension (Olympus). Experiments were performed applying a standard protocol. First, the fluorescence background of the uncontaminated glass support was determined. Then ca. 20 μL of protein solution (0.5 mg of fluorophore-labeled protein in 1 mL of freshly prepared phosphate buffered saline, PBS) were placed on the glass. After 2 min, the substrate was rinsed for 8 s with PBS, subsequently for 8 s with Millipore water, and finally dried in a gentle nitrogen stream. Then, the fluorescence intensity of the protein exposed sample was determined. Adsorption experiments were performed at least thrice for all proteins, with a minimum of six pictures taken at different positions of each sample. The relative amounts of adsorbed protein determined represent arithmetic averages of the fluorescence intensities after subtracting the background.

## 3. Results and Discussion

### 3.1. Monomer and Polymer Synthesis

As described for monomers 1c, 1d, 3c, 3d, 6c, and 6d [50], the new monomers 2c, 2d, 4c, 4d, 5c, and 5d were prepared by reaction of the respective tertiary amine functionalized methacrylates with cyclic sulfate diesters in acetonitrile, namely with ethylene sulfate or propylene sulfate. The methacrylate intermediates bearing tertiary amines were synthesized by transesterification of methyl methacrylate with the corresponding amino alcohols [38]. In all cases, a 10% molar excess of the tertiary amine intermediate with respect to the alkylating agent was employed, and rather long reaction times were used, in order to assure maximum consumption of the cancerogenic cyclic sulfates. As the zwitterionic products precipitate readily from the reaction solution, their separation and purification is straightforward. The analytical data (from elemental analysis, mass spectrometry, ^1^H-NMR and ^13^C-NMR spectroscopy, infrared spectroscopy) confirmed the successful synthesis and the purity of the products.

Via free radical polymerization, the various sulfabetaine monomers were smoothly converted into their homopolymers. Polymerizations were conducted in homogeneous solution in trifluoroethanol (TFE), which is one of the few effective solvents for most zwitterionic monomers and their polymers [30,38,54]. All polymers were modestly hygroscopic and colorless solids. Key analytical data of the homopolymers are summarized in Table 2. The apparent molar masses of the various polymers were rather high, being in the range of 130 to 420 kg mol^−1^. Polymer dispersities *Ð* were in the range of ~2 to ~4, in agreement with a standard free radical polymerization process conducted up to high yields. 

### 3.2. General Properties of the Homopolymers 

**Figure 2:**
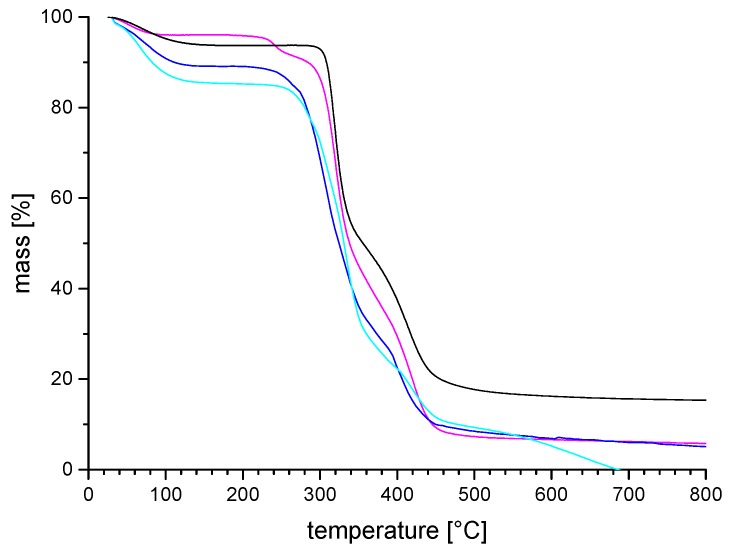
Exemplary TGA thermograms of zwitterionic polymer series P-4: (−−−) = P-4a; (−−−) = P-4b; (−−−) = P-4c; (−−−) = P-4d.

The thermal behavior of the poly(sulfabetaine)s in bulk was studied by thermogravimetric analysis (TGA) and differential scanning calorimetry (DSC). TGA of the hygroscopic polymers under inert gas revealed three temperature regimes of weight loss (Figure 2). When the samples had been previously handled in air, bound water was lost at temperatures between 80 up to 150 °C, typically amounting to about 5–10 wt % for the poly(sulfabetaine)s. This weight loss is reversible, and corresponds to approximately one water molecule per constitutional repeat unit (CRU). This first weight loss seems somewhat less pronounced for the poly(sulfabetaine)s than for their sulfobetaine analogues, suggesting a lower hygroscopy of the former polyzwitterion class. Next, an irreversible step of weight loss is observed due to thermal decomposition starting at 250 °C, accounting for a mass loss of about 40–50 wt %, followed by a third step starting at about 330 °C. Accordingly, the thermal stability of the poly(sulfabetaine)s is comparable to the one of their sulfobetaine analogues [33,38], as illustrated in Figure 2. Putatively, the second step may be attributed to a dealkylation step at the nitrogen atom of the betaine moieties, the alkylsulfate groups being eliminated, as demonstrated for other polyzwitterions such as poly(carboxybetaine)s previously [22]. Thermograms of dried samples recorded by differential scanning calorimetry (DSC) did not exhibit thermal transitions for any of the poly(sulfabetaine)s before degradation starts, concordantly with reports on most poly(sulfobetaine)s [33,38,55].

Solubility of the poly(sulfabetaine)s was generally low. They were insoluble in most organic solvents, including highly polar ones such as dimethylsulfoxide, acetonitrile, *N,N*-dimethyl-formamide, *N,N*-dimethylacetamide, or *N*-methylpyrrolidone. They were also insoluble in the protic polar solvent formamide HCONH_2_, which is known to dissolve many poly(sulfobetaine)s [32,33]. Most suited solvents were TFE, HFIP, and trifluoroacetic acid, which are typically considered to be good solvents for polyzwitterions in general [30,32,33,54,56]. Still, the solubilities of the polystyrene-based poly(sulfobetaine)s P−6c and P−6d were so low that the high molar mass samples produced swelled strongly in TFE or HFIP, but dissolved only sparingly.

The rather high molar masses of the synthesized poly(sulfabetaine)s aggravated the problem of the generally low solubility of most polyzwitterions in pure water. While the poly(sulfobetaine) analogues P−1a and P−3a that are used as references show a miscibility gap in pure aqueous solution at lower temperatures, i.e., UCST behavior, but are soluble in warm and hot water [38,57], none of the poly(sulfabetaine)s prepared could be dissolved in pure water up to the boiling point. Only when substantial amounts of inorganic salts were added, these polymers became water-soluble (Table 2). The “salting-in” phenomenon is well known for many polyzwitterions [34,38,58,59,60,61,62,63]. Still, in contrast to their sulfobetaine analogues, which all dissolve in normal saline solution (“physiological saline”, 9.0 g/L NaCl) at ambient temperature [38], polymers P−1c to P−6c and P−1d to P−6d require much higher concentrations of added salt to form aqueous solutions, corroborating the few literature reports existing [43,47,49,51]. Consistently, none of the poly(sulfabetaine)s dissolved in PBS buffer. The strongly reduced water-solubility of poly(sulfabetaine)s compared to their sulfobetaine analogues is in agreement with molecular modeling studies deducing lower hydrophilicity for the ammoniosulfate in comparison to the ammoniosulfonate moiety [46,64]. The solubilities of the morpholine-derived poly(sulfabetaine)s P−5c and P−5d, and of the polystyrene-based poly-(sulfabetaine)s P−6c and P−6d were so low that the high molar mass samples used only swelled in brine.

### 3.3. Preparation of Thin Hydrogel Coatings

**Figure 3:**
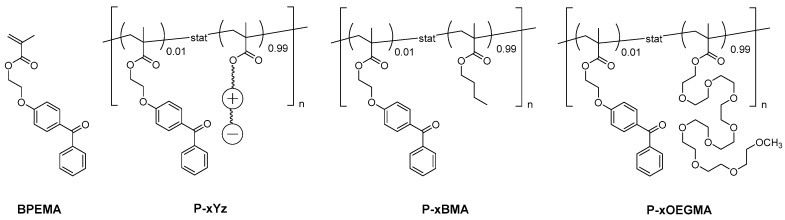
Chemical structure of the photo-reactive comonomer BPEMA used, the photo-crosslinkable copolymers P–xYz (with Y = 1−6 and z = a−d; see also Figure 1), and of the reference copolymer systems P–xBMA and P–xOEGMA.

Similarly to homopolymer synthesis, free radical copolymerization of the sulfabetaine monomers together with 1 mol % of comonomer BPEMA (Figure 3) smoothly produced the corresponding copolymers in high yields. Copolymer P−x1d and reference copolymers P−x1a (“P−xSPP”), P−x3a (“P−xSPE”), P−xOEGMA, and P−xBMA (cf. Figure 1 and Figure 3) were made analogously before, as reported elsewhere [52]. 

Key analytical data of the photo-crosslinkable copolymers are summarized in Table 3. The apparent number average molar masses *M*_n_^app^ of the various polymers were in the range of 100 to 250 kg mol^−1^, i.e., they were similar, though slightly smaller than the molar masses of the homopolymers. Similarly, the copolymer dispersities *Ð* were in the range of 2.8–4. The photo-crosslinker content in the copolymers was determined to be about 1 mol % (by ^1^H NMR, based on CRU), corresponding within the limits of precision to the content of monomer BPEMA in the copolymerization mixture. Considering that the molar masses of the zwitterionic monomers are in the range of 300 to 340 Da, these data suggest that the copolymer chains incorporate on average four to seven photo-reactive benzophenone moieties. 

The solubility of the various poly(sulfabetaine)s in aqueous media did not virtually differ from the behavior of their homopolymer analogues, i.e., they required high concentrations of added salt, the concentration of 9 g L^−1^ of NaCl of normal saline not being sufficient. The copolymers seemed to dissolve somewhat more readily in TFE than the corresponding homopolymer samples. Yet, it is currently not clear whether this is due to the (at least apparently) slightly reduced molar masses, or due to the comonomer content. Still, the polystyrene derivatives P−6c and P−6d, and also samples P−4d and P−5c did not dissolve sufficiently in TFE and HFIP to allow for SEC analysis, or for the spin-coating preparations (see below).

**Figure 4:**
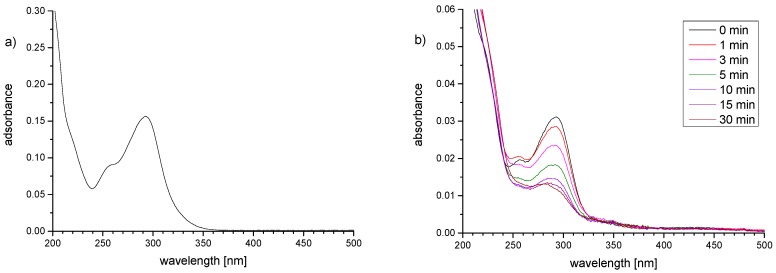
(**a**) UV-Vis spectra of photo-reactive monomer BPEMA in TFE solution (0.01 mM); (**b**) UV-Vis spectra of hydrogel films of P−x3a (P−xSPE) on a quartz support as function of the exposure-time to UV-irradiation (from top to bottom: 0, 1, 3,5, 10, 15, and 30 min).

The sufficiently soluble copolymers were spin-coated from TFE solutions onto glass slides, which beforehand had been modified by an aminosilane monolayer (water contact angle 59 ± 4°), to yield very smooth pinhole-free, coherent, and homogeneous films, with RMS roughnesses below 0.4 nm as exemplified for P−x1a, P−x1d, P−x3a, and P−xOEGA by AFM [52]. Exceptionally, reference P−xBMA was spin-coated from ethyl acetate solution, as films prepared from TFE solutions were quite rough [52]. The spin-coated films were stabilized by irradiation with near-UV light (Figure 4). This gives rise to simultaneous covalent cross-linking of the chains and covalent attachment to the support due to the specific photochemical reactivity of the benzophenone moiety [52,53,65,66,67,68,69,70,71], overcoming the need for incorporating two different reactive groups in the hydrogel film forming polymer [72,73]. With its spectral cut-off at 310 nm, the irradiation system chosen excites only the flank of the intense absorbance band centered at about 295 nm of the 4-alkoyxy benzophenone chromophore (Figure 4a). This favors the in-depth homogeneous curing of the films, as the light intensities at the top and the bottom of the film are comparably high due to the weak absorbance. 

Figure 4b illustrates that the photoreaction is fast, approaching its maximum after about 15 min. The decreasing intensity of the 295 nm band is attributed to the increasing consumption of the carbonyl group by H-abstraction, interrupting the conjugated π-system of the benzophenone ether chromophore. Further irradiation reduced the remaining absorbance only marginally. As prolonged irradiation risks to favor competing nonspecific photo-degradation reactions, an irradiation time of 30 min was considered to be optimal under the conditions employed, and thus chosen for the photo−curing of all samples.

**Figure 5:**
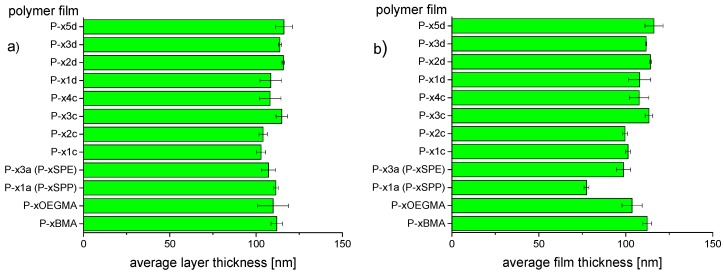
Ellipsometric thickness of dry polyzwitterion films: (**a**) measured after photo-curing, and (**b**) measured after the subsequent washing procedure.

The conditions for spin-coating were slightly adapted for each polymer to obtain consistently film thicknesses of about 100 nm (Figure 5). This value appeared a reasonable choice, as very thin hydrogel coatings (<30 nm) seem prone to enhanced fouling [74,75]. Still, reports are somewhat conflicting with respect to the existence and range of an optimal thickness of thin hydrogel films for reducing protein fouling [76,77,78,79,80,81]. Hence in prior exploratory experiments with reference copolymer P−x3a (P−xSPE), we clarified that protein adsorption increased notably when the thickness of hydrogel films dropped below 80 nm, whereas the adsorbed amounts were about the same for thicknesses up to 120 nm at least (vide infra, see also Figure 7a below).

By the irradiation, the polymer films were effectively fixed on the supports. In most cases, even extended washing reduced film thickness hardly, if at all, within the precision of the measurements (Figure 5b). Comparatively high losses (ca. 25%) were exceptionally encountered for reference copolymer P−x1a (P−xSPP), which is in fact the most water-soluble polymer of the series studied next to reference copolymer P−xOEGMA. The latter however, may be more crosslinked than the zwitterionic polymers, as it is prone to photo-crosslinking under ambient conditions (air) even in the absence of benzophenone groups [53,82]. This is explained by the numerous α-methylene ether hydrogen atoms contained, which are most easily abstracted by oxygen centered radicals. Importantly, the findings demonstrate that the a priori low content of photo-reactive benzophenone moieties is sufficient to produce stable coatings from all polymers studied. Still, the extent of overall cross-linking must be assumed to be low. This implies that the hydrogel films produced are well swellable in aqueous media on the one hand, but mechanically very soft on the other hand.

**Figure 6:**
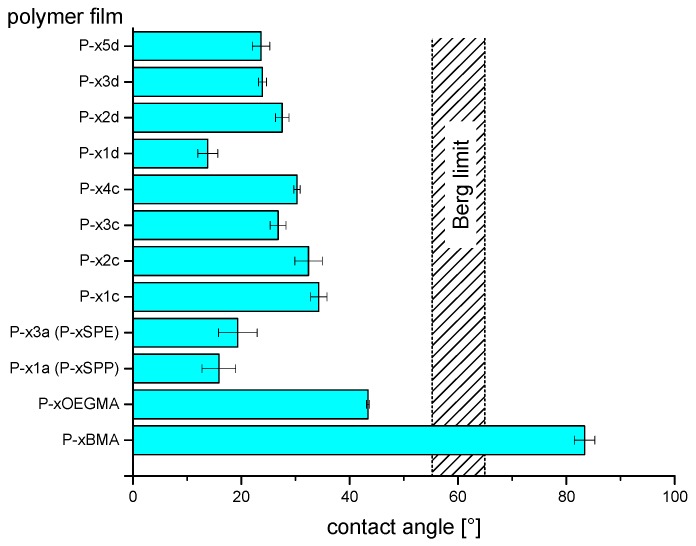
Water-air contact angles measured for glass supports coated with various polyzwitterions and reference polymers.

The spin-coated films were analyzed by optical microscopy and water-air contact angle measurements (Figure 6). Films were coherent, smooth, and free of defects within optical resolution. Water-air contact angle (WCA) measurements showed that only the coatings made from reference P−xBMA give rather hydrophobic surfaces (WCA~80°), as may have been anticipated. The WCA values of all other films are below the so-called “Berg limit” of about 60° for spontaneous hydrophobic fouling [83,84,85].

Within the various polyzwitterion coatings, the films of the sulfobetaine polymers employed for comparison, namely of P−x1a (P−xSPP) and P−x3a (P−xSPE), showed WCAs of about 15−20° in agreement with a previous study [52]. The new poly(sulfabetaine) coatings had also very hydrophilic surfaces, though they exhibited typically somewhat higher WCAs of around 25° with only small differences between the samples. The higher WCA values of the poly(sulfabetaine) coatings may reflect the lower solubility of the underlying polymers in aqueous media in comparison to their sulfobetaine analogs (see above). In contrast, films of the nonionic reference P−xOEGMA exhibit a considerably higher contact angle of about 40°, which is still well below the Berg limit. 

### 3.4. Resistance Against Unspecific Adsorption of Proteins (“Fouling”)

**Figure 7:**
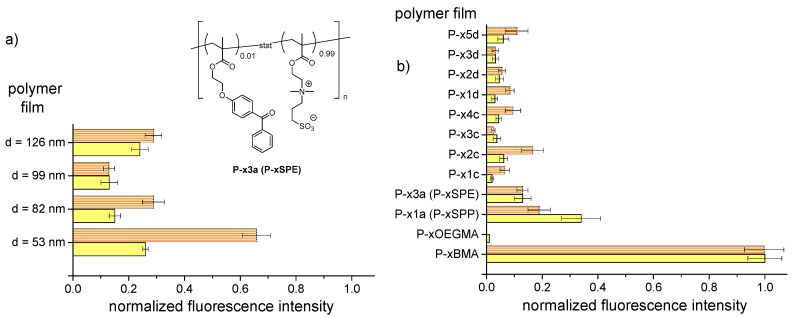
Adsorption of fluorophore-labeled proteins fibrinogen (orange bars) and BSA (yellow bars) from PSB solution onto polyzwitterion hydrogel coatings, according to the sample’s fluorescence intensity after exposure (intensities normalized to the intensities measured for P−xBMA): (**a**) Upon variation of the hydrogel film thickness for P−x3a (P−xSPE); (**b**) upon variation of the polyzwitterion structure, while keeping film thickness at about 100 nm. Films of the nonionic P−xOEGMA and the hydrophobic P−xBMA are used as references (positive and negative control, respectively).

The effectiveness of the poly(sulfobetaine) films to reduce protein fouling was screened using two fluorophore—labeled model proteins, namely bovine serum albumin (BSA) and fibrinogen. For that, samples were exposed to solutions containing the protein in PBS buffer (pH = 7.4) for a specific time, then rinsed by PBS and water to remove the residual protein solution as well as loosely adsorbed protein, and dried, before the relative amount of irreversibly adsorbed protein was quantified by measuring the fluorescence intensity. Samples coated by the hydrophobic P−xBMA and hydrophilic P−xOEGMA polymers served as negative and, respectively, positive controls. Further, coatings of the often-used zwitterionic polymers P−x1a (“P−xSPP”) and P−x3a (“P−xSPE”) were included for comparison. Under these conditions, the amphiphilic BSA, which also tends to adsorb due to hydrophobic interactions, is markedly negatively charged (IEP~4.8), while fibrinogen is moderately negatively charged (IEP~5.9) [86,87]. The results of the experiments are displayed in Figure 7.

Already on a first view, Figure 7 shows that all polyzwitterions coatings strongly reduced the adsorption of both BSA and fibrinogen compared to the negative control P−xBMA. It was also evident from Figure 7b that their anti-fouling capabilities were somewhat lower than that of the positive control of P−xOEGMA. Nevertheless, the results demonstrate that not only the well-established polyzwitterions of the sulfobetaine type (as references P−xSPP and P−xSPE) but also such of the sulfabetaine type can confer distinct low-fouling behavior to surfaces. 

Figure 7a illustrates for polymer P−x3a (P−xSPE) the influence of the hydrogel film thickness on the fouling resistance. While the thinnest film of about 50 nm thickness is somewhat less resistant to protein fouling, in particular to fibrinogen, than the thicker ones, the latter hydrogel films appear be comparatively effective in repelling proteins. The data might suggest a shallow minimum of fouling for films that are approximately 100 nm thin, but due to the limited experimental statistics of this explorative study, this observation cannot be considered significant. In any case, when studying the effect of structure variation on fouling efficacy, we used uniformly coatings of ~100 nm thickness for all polymers (Figure 7b).

Analyzing Figure 7b in more detail, additional features can be derived. We note that in agreement with a previous study using surface plasmon resonance SPR [52], the anti-fouling effectivity of the poly(sulfobetaine)s P−x1a and P−x3a (P−xSPP and P−xSPE) used as references were equal against fibrinogen, while P−x1a was more efficient than P−x3a in repelling BSA.

In comparison to the references, all poly(sulfabetaine) coatings tested were similarly or even more effective in reducing protein fouling. In particular the adsorption of BSA was notably lower for the poly(sulfabetaine) films than for the sulfobetaine references, which are already highly fouling-resistant. This finding was unexpected, as under the test conditions (namely in PBS buffer), the poly(sulfabetaine)s are not water-soluble in contrast to their sulfobetaine analogues (see Table 3), and are thus less hydrated. Moreover, theoretical studies predicted the contrary performance [64]. When comparing the protein fouling resistance of the poly(sulfabetaine) series c and d, efficacies were about equal. This implies that the precise length of the spacer group separating the cationic and the anionic moieties, namely a dimethylene or a trimethylene spacer, is of little importance for their anti-fouling effect. Furthermore, the findings corroborate that the relative anti-fouling efficiencies of the individual polymers differ for individual proteins [12]. There is no absolute “champion”, but the detailed performance depends on the specific pair of polyzwitterion and protein. For instance, highest fouling resistance against BSA was encountered for coatings of P−x1c, which gives nearly the same excellent result as the control P−xOEGMA. In contrast, highest fouling resistance against fibrinogen was observed for films of P−x3c. The best combination of fouling resistance against both proteins was found for P−x3c and P−x3d. Still, one should not overstress these rankings, as the anti-fouling effects of all the polyzwitterions were generally high and the observed differences were comparatively small.

## 4. Conclusions

A large variety of poly(sulfabetaine) methacrylic monomers is easily accessible by ring-opening alkylation of polymerizable tertiary amines with cyclic sulfates. Their straightforward free radical polymerization provides high molar mass polymers in good yields. These polyzwitterions are insoluble in most solvents, including pure water, physiological saline, and PBS buffer. Still, solubility in aqueous media can be achieved in many cases by the addition of high concentrations of inorganic salts such as NaCl. When copolymerized with small amounts of benzophenone bearing monomers, the copolymers formed can be spin-coated and photo-crosslinked to yield stable thin hydrogel coatings. An exploratory screening demonstrated that such coatings are highly resistant against the nonspecific adsorption of proteins. While their detailed anti-fouling performance depends on the precise couple protein−polyzwitterion, it tends to surpass even the one of films prepared from poly(sulfobetaine) analogues, which have been established as effective fouling protection coatings. As previous studies have also shown good long-term stability of polysulfabetaines against hydrolysis in aqueous media, except for very low or high pH values where polysulfobetaines excel [50], such polymers may be useful components for implementing low-fouling behavior to surfaces.

## Figures and Tables

**Table 1:** Reaction conditions for the homo- and copolymerization of the sulfabetaine monomers in trifluoroethanol (TFE) solution (60 °C, 20 h, 20 wt % solids), using initiator AIBN and crosslinker BPEMA.

Polymer	Monomer (mg)	Monomer (mmol)	Initiator (mg)	Initiator (mmol)	Crosslinker (mg)	Crosslinker (mmol)
**P−x1c**	1180	4.00	3.3	0.02	12.4	0.04
**P−2c**	300	1.00	0.8	0.005	0	-
**P−x2c**	1770	6.00	4.9	0.03	18.6	0.06
**P−x3c**	1130	4.00	3.3	0.02	12.4	0.04
**P−4c**	670	2.00	1.6	0.01	0	-
**P−x4c**	3210	10.0	8.2	0.05	31.0	0.1
**P−5c**	650	2.00	1.6	0.01	0	-
**P−x5c**	1620	5.00	4.1	0.025	15.5	0.05
**P−x6c**	1140	4.00	3.3	0.02	12.4	0.04
**P−2d**	620	2.00	1.6	0.01	0	-
**P−x2d**	2470	8.00	6.6	0.04	24.8	0.08
**P−x3d**	5910	20.00	16.4	0.1	62.0	0.2
**P−4d**	670	2.00	1.6	0.01	0	-
**P−x4d**	3350	10.00	8.2	0.05	31.0	0.1
**P−5d**	640	2.00	1.6	0.01	0	-
**P−x5d**	640	2.00	1.6	0.01	6.2	0.02
**P−x6d**	1200	4.00	3.3	0.02	12.4	0.04

**Table 2:** Molar masses of the homopolymers prepared, and their solubility (3 wt %) in various aqueous solvents.

Polymer	Yield [%]	*M*_n_^app^ [kg mol^−1^] ^(a)^	Dispersity *Ð* ^(a)^	*T*_decomp_ [°C] ^(b)^	CP in PBS [°C] ^(c)^	CP in Brine [(°C] ^(d)^
**P−1c**	36	- ^(e)^	- ^(e)^	290	- ^(f)^	<0
**P−1d**	34	220	2.3	290	- ^(f)^	<0
**P−2c**	87	- ^(e)^	- ^(e)^	290	- ^(f)^	sw ^(g)^
**P−2d**	46	180	3.3	290	- ^(f)^	<0
**P−3c**	82	400	1.8	280	- ^(f)^	<0
**P−3d**	85	420	1.6	290	- ^(f)^	<0
**P−4c**	89	220	3.2	290	- ^(f)^	<0
**P−4d**	89	- ^(e)^	- ^(e)^	270	- ^(f)^	sw ^(g)^
**P−5c**	92	- ^(e)^	- ^(e)^	270	- ^(f)^	sw ^(g)^
**P−5d**	75	- ^(e)^	- ^(e)^	270	- ^(f)^	sw ^(g)^
**P−6c**	quant.	- ^(e)^	- ^(e)^	290	- ^(f)^	sw ^(g)^
**P−6d**	quant.	- ^(e)^	- ^(e)^	290	- ^(f)^	sw ^(g)^

^(a)^ apparent number average molar mass *M*_n_^app^ and dispersity *Ð* (*M*_w_^app^/*M*_n_^app^) by size exclusion chromatography (SEC), eluent HFIP containing 50 mM of CF_3_COONa, calibration with linear poly(methyl methacrylate) standards; ^(b)^ onset by TGA in nitrogen atmosphere, heating rate 10 K min^−1^; ^(c)^ cloud point in phosphate buffered saline; ^(d)^ cloud point in saturated aqueous NaCl solution; eluent NMP; ^(e)^ insoluble in eluent.; ^(f)^ not soluble up to 100 °C; ^(g)^ swollen in solvent.

**Table 3:** Molar masses of the cross-linkable copolymers prepared.

Polymer	Yield [%]	*M*_n_^app^ [kg mol^−1^] ^(a)^	Dispersity *Ð* ^(a)^	Content of BPEMA [mol %] ^b)^	Soluble in TFE
**P−x1c**	45	- ^(c)^	- ^(c)^	1.5 ± 0.5	yes
**P−x1d**	79	190	3.6	1.1 ± 0.5	yes
**P−x2c**	54	130	3.7	1.1 ± 0.5	yes
**P−x2d**	76	130	3.8	1.4 ± 0.5	yes
**P−x3c**	90	>240 ^(d)^	>2.8 ^(d)^	1.1 ± 0.5	yes
**P−x3d**	91	>230 ^(d)^	>3.0 ^(d)^	0.8 ± 0.5	yes
**P−x4c**	quantitative	140	4.1	0.8 ± 0.5	yes
**P−x4d**	quantitative	- ^(c)^	- ^(c)^	0.9 ± 0.5	sw ^(f)^
**P−x5c**	89	- ^(c)^	- ^(c)^	1.0 ± 0.5	sw ^(f)^
**P−x5d**	76	110	4.4	1.3 ± 0.5	yes
**P−x6c**	quantitative	- ^(c)^	- ^(c)^	n.d. ^(e)^	sw ^(f)^
**P−x6d**	quantitative	- ^(c)^	- ^(c)^	n.d ^(e)^	sw ^(f)^
**P−x1a(P–xSPP)**	56	120	5.0	1.2 ± 0.5	yes
**P−x3a(P–xSPE)**	63	90	5.4	1.1 ± 0.5	yes
**P−xOEGMA**	82	130	3.7	1.0 ± 0.5	yes
**P−xBMA**	53	50	2.8	1.3 ± 0.5	yes

^(a)^ apparent number average molar mass *M*_n_^app^ and dispersity *Ð* (*M*_w_^app^/*M*_n_^app^) by SEC, eluent HFIP containing 50 mM of sodium trifluoroacetate, calibration with linear poly(methyl methacrylate) standards; ^(b)^ by ^1^H NMR; ^(c)^ not sufficiently soluble in eluent.; ^(d)^ parts of the sample approach exclusion volume; ^(e)^ not determined, signals of cross-linker not resolved in spectrum; ^(f)^ strongly swollen, though not fully soluble.

**Figure A1:**
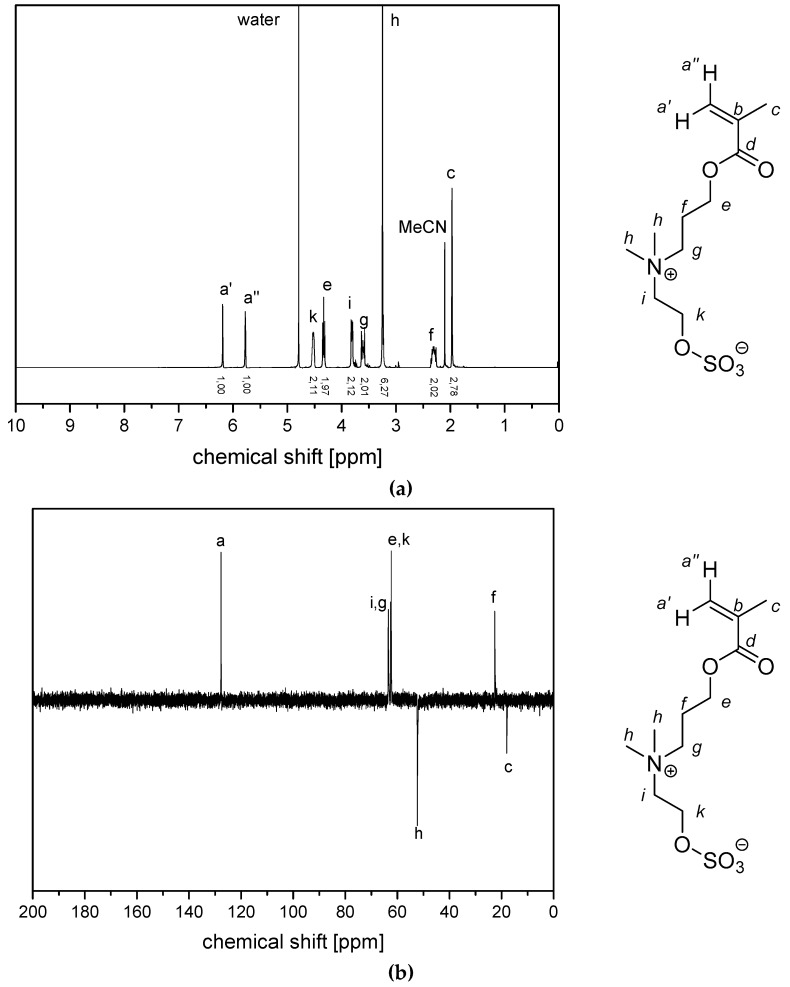
(**a**) ^1^H NMR- spectra and (**b**) ^13^C (APT) NMR-spectra of monomer 2c in D_2_O.

**Figure A2:**
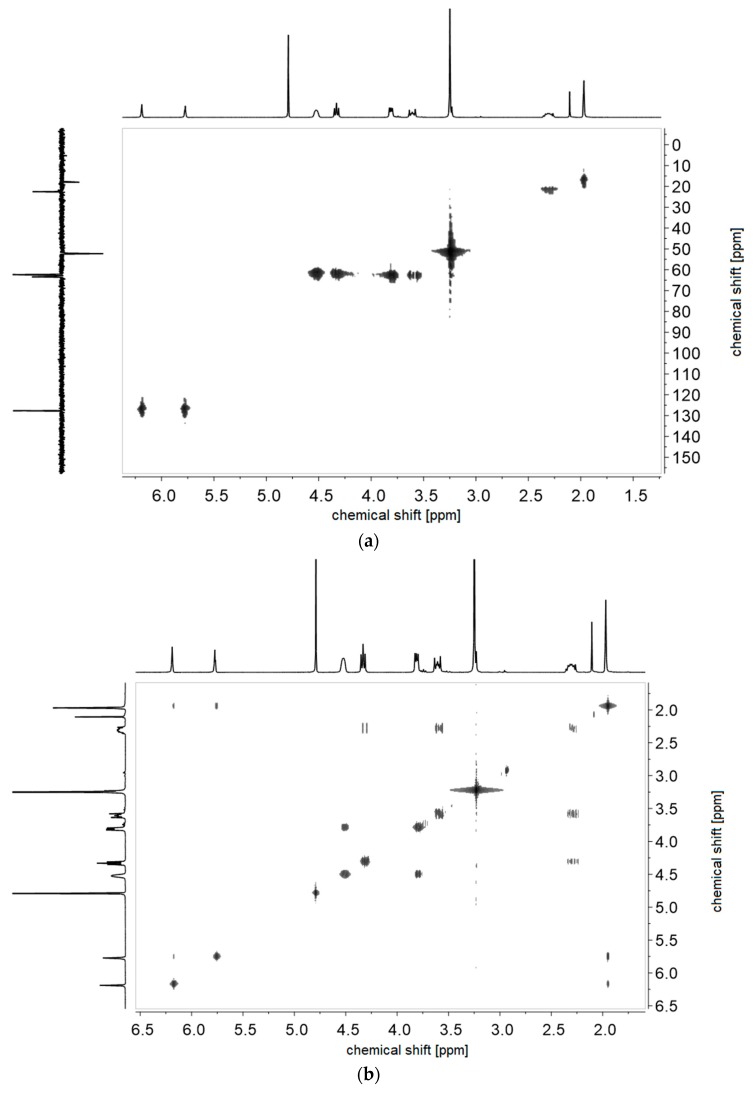
(**a**) ^1^H-^1^H-COSY- and (**b**) ^1^H-^13^C-HMQC NMR-spectra of monomer of 2c in D_2_O.

**Figure A3:**
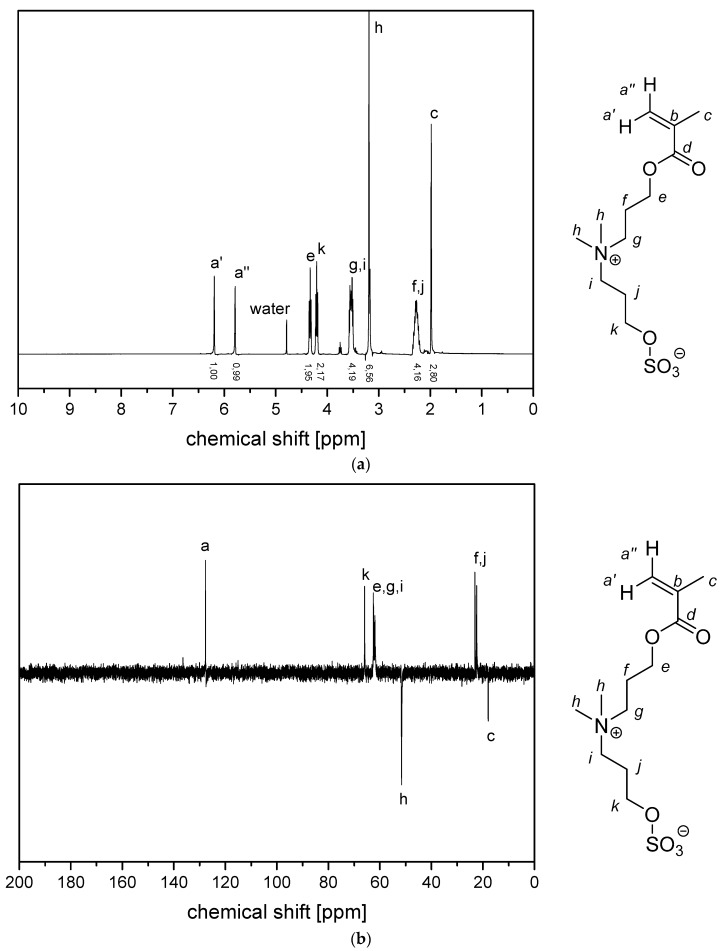
(**a**) ^1^H NMR- spectra and (**b**) ^13^C (APT) NMR-spectra of monomer 2d in D_2_O.

**Figure A4:**
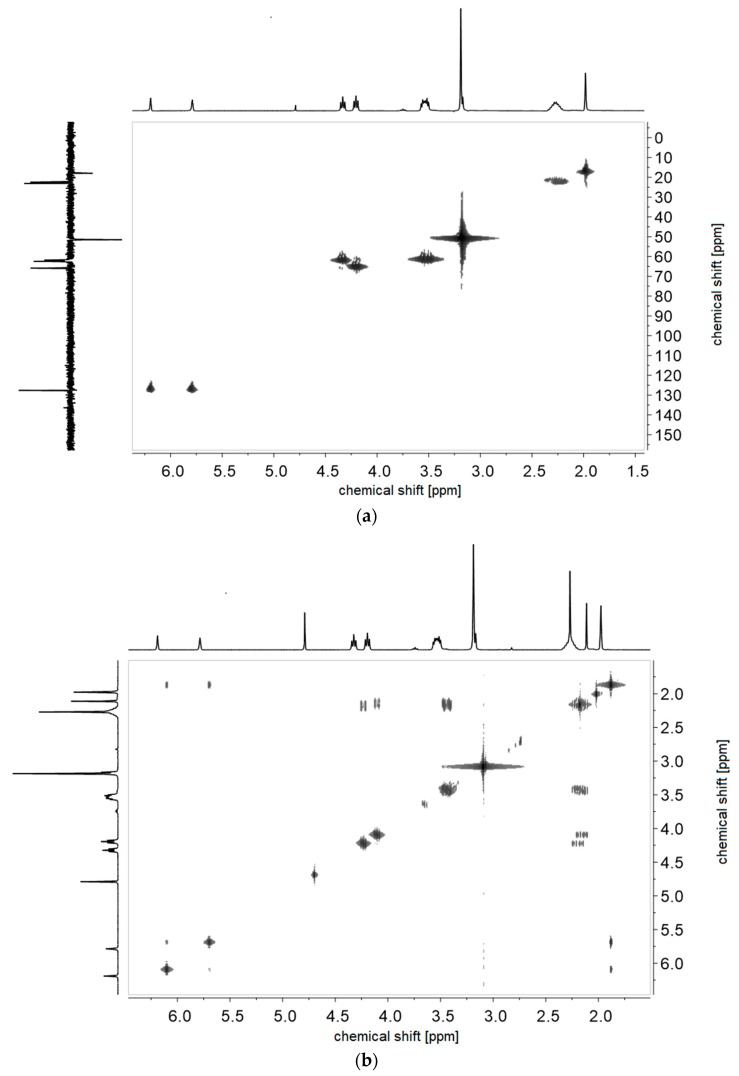
(**a**) ^1^H-^1^H-COSY- and (**b**) ^1^H-^13^C-HMQC NMR-spectra of monomer of 2d in D_2_O.

**Figure A5:**
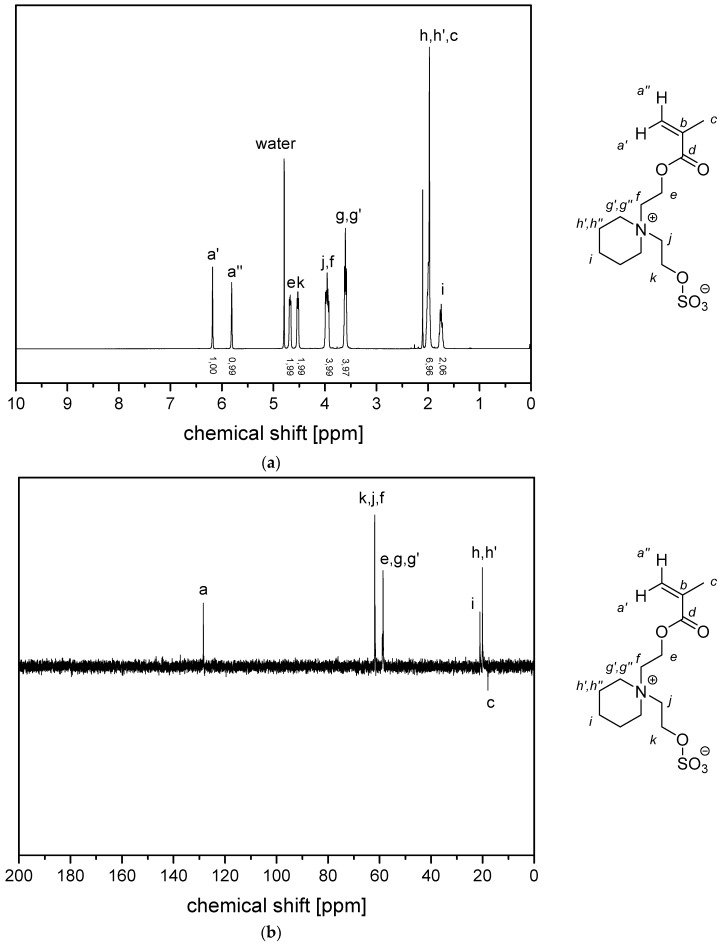
(**a**) ^1^H NMR- spectra and (**b**) ^13^C (APT) NMR-spectra of monomer 4c in D_2_O.

**Figure A6:**
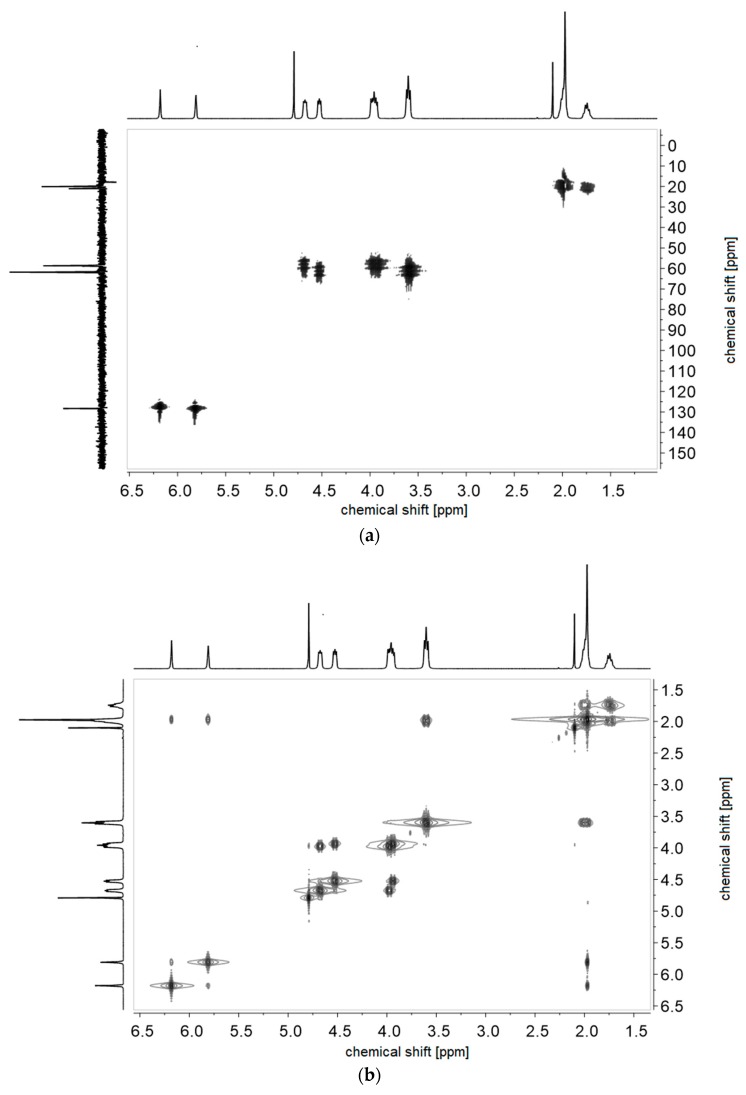
(**a**) ^1^H-^1^H-COSY- and (**b**) ^1^H-^13^C-HMQC NMR-spectra of monomer of 4c in D_2_O.

**Figure A7:**
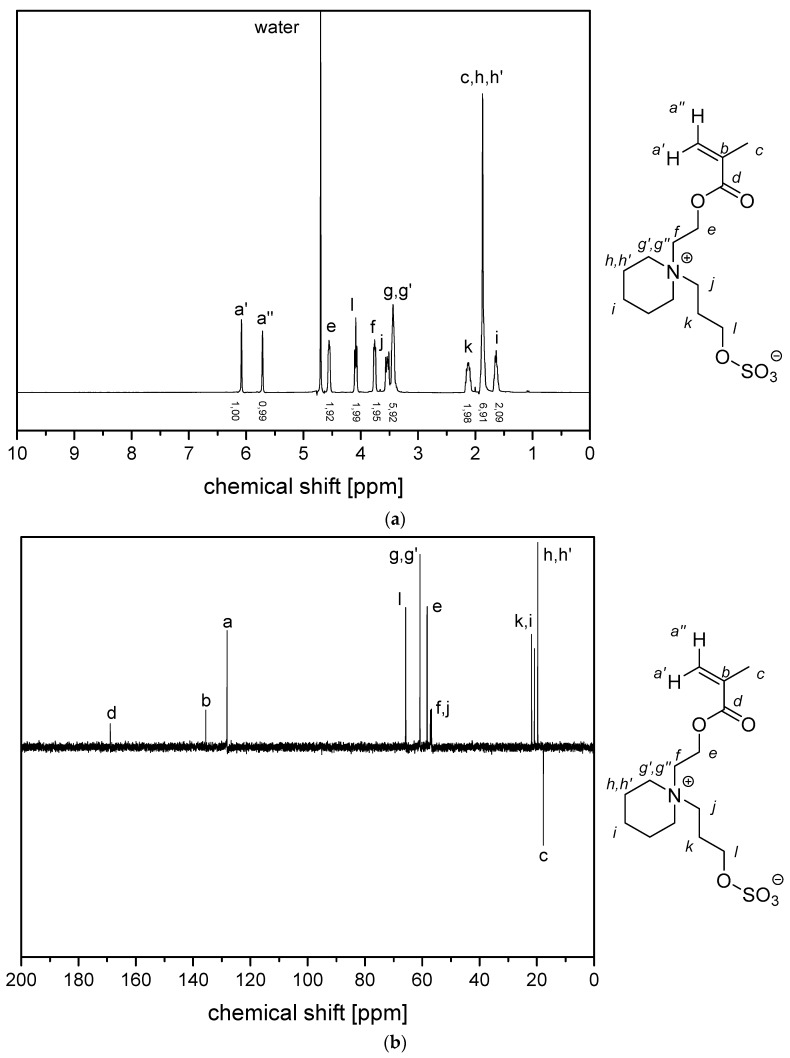
(**a**) ^1^H NMR- spectra and (**b**) ^13^C (APT) NMR-spectra of monomer 4d (in D_2_O).

**Figure A8:**
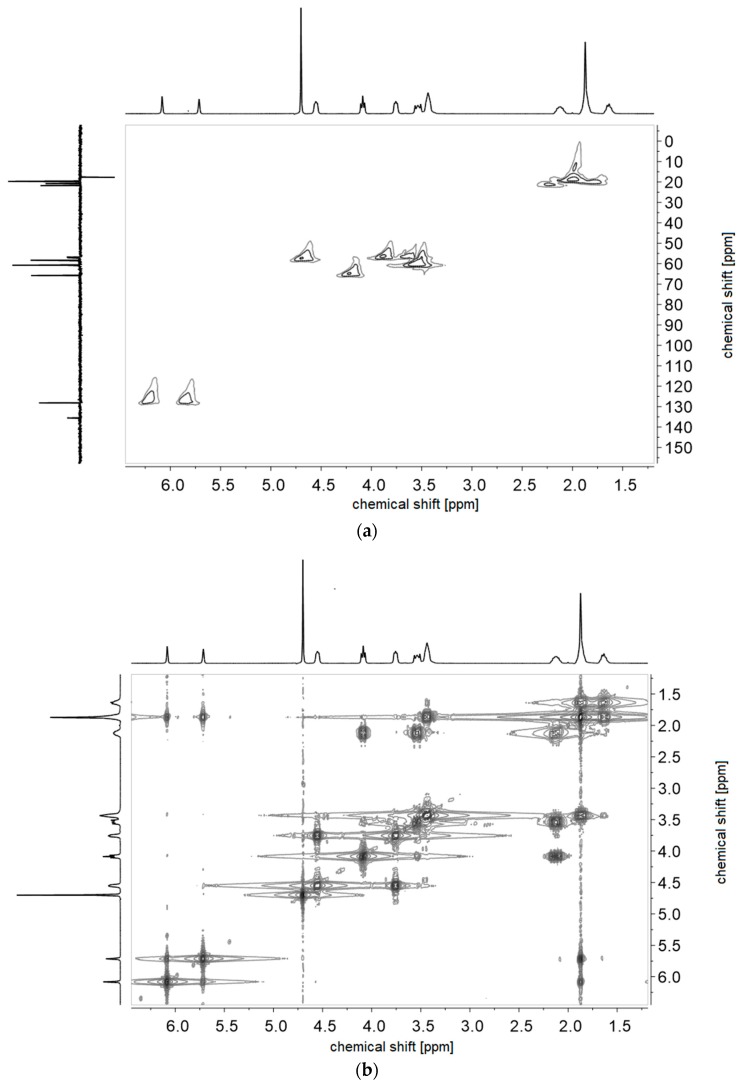
(**a**) ^1^H-^1^H-COSY- and (**b**) ^1^H-^13^C-HMQC NMR-spectra of monomer of 4d in D_2_O.

**Figure A9:**
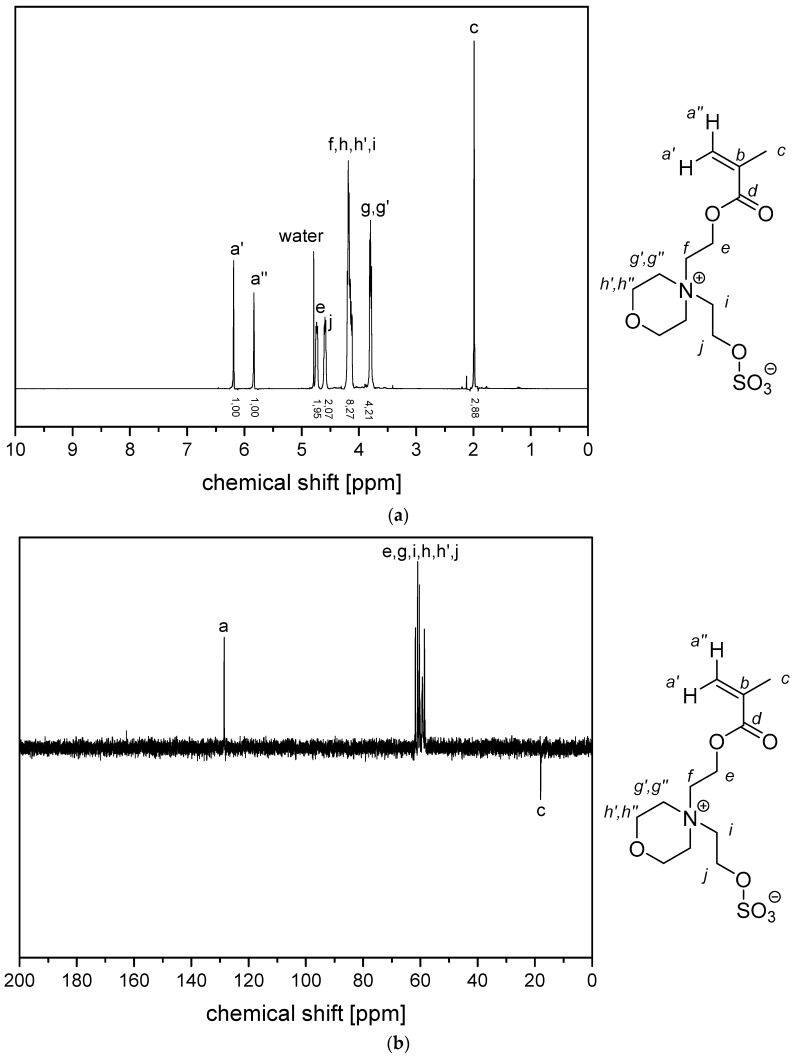
(**a**) ^1^H NMR-spectra and (**b**) ^13^C (APT) NMR-spectra of monomer 5c in D_2_O.

**Figure A10:**
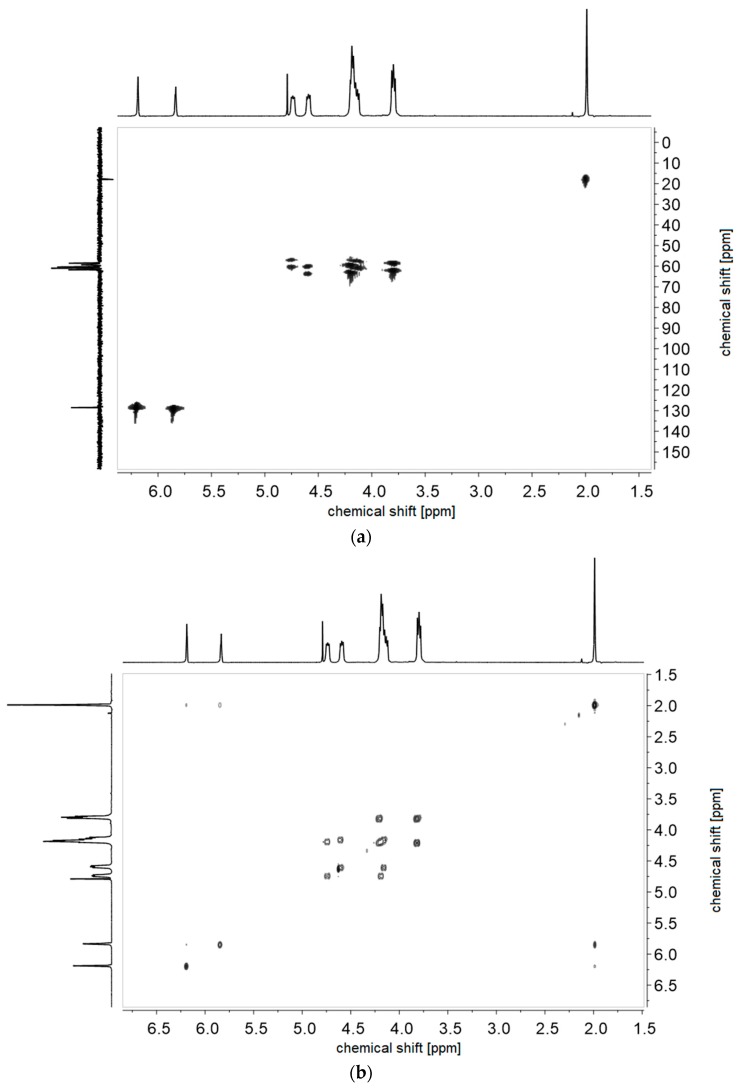
(**a**) ^1^H-^1^H-COSY- and (**b**) ^1^H-^13^C-HMQC NMR-spectra of monomer of 5c in D_2_O.

**Figure A11:**
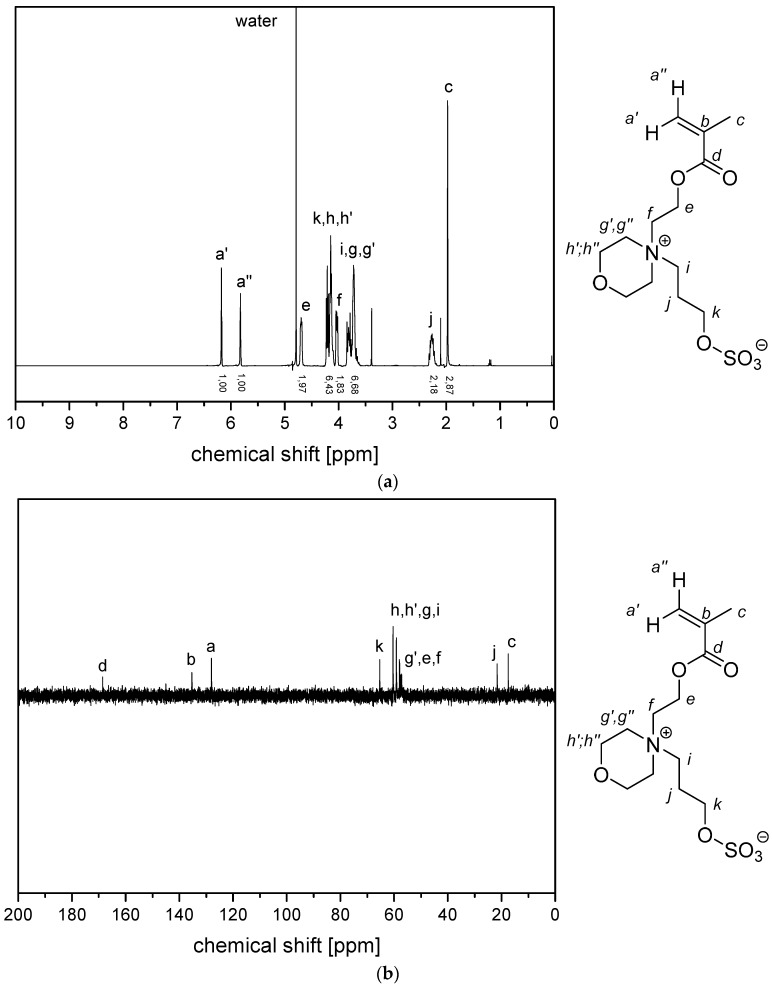
(**a**) ^1^H NMR- spectra and (**b**) ^13^C (APT) NMR-spectra of monomer 5d in D_2_O.

**Figure A12:**
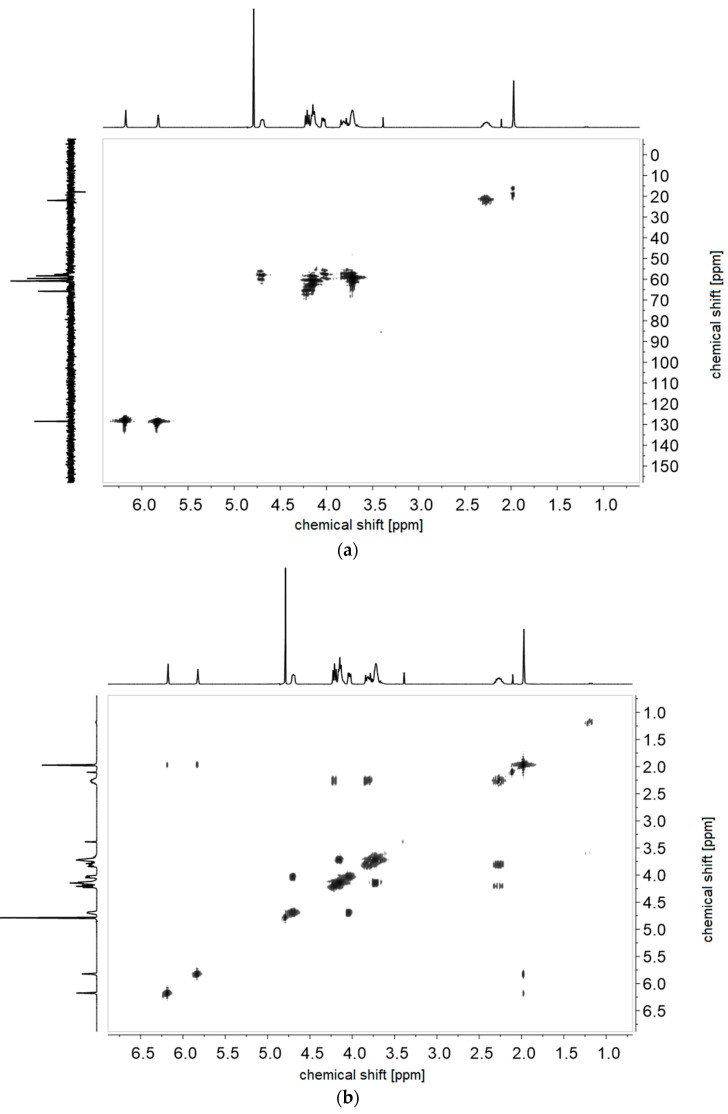
(**a**) ^1^H-^1^H-COSY- and (**b**) ^1^H-^13^C-HMQC NMR-spectra of monomer of 5d in D_2_O.

## Appendix A

^1^H NMR- and ^13^C (APT) NMR-spectra of monomer sand polymers in aqueous solution.

## References

[1] Galin J.-C., Salamone J.C. (1996). Polyzwitterions. Polymer Materials Encyclopedia.

[2] Lowe A.B., McCormick C.L. (2002). Synthesis and solution properties of zwitterionic polymers. Chem. Rev..

[3] Laschewsky A. (2014). Structures and Synthesis of Zwitterionic Polymers. Polymers.

[4] Chapman D. (1993). Biomembranes and new hemocompatible materials. Langmuir.

[5] Chen M., Briscoe W.H., Armes S.P., Klein J. (2009). Lubrication at physiological pressures by polyzwitterionic brushes. Science.

[6] Schlenoff J.B. (2014). Zwitteration: Coating Surfaces with Zwitterionic Functionality to Reduce Nonspecific Adsorption. Langmuir.

[7] Ilčíková M., Tkáč J., Kasák P. (2015). Switchable Materials Containing Polyzwitterion Moieties. Polymers.

[8] He M., Gao K., Zhou L., Jiao Z., Wu M., Cao J., You X., Cai Z., Su Y., Jiang Z. (2016). Zwitterionic materials for antifouling membrane surface construction. Acta Biomater..

[9] Biehl P., von der Lühe M., Dutz S., Schacher F.H. (2018). Synthesis, Characterization, and Applications of Magnetic Nanoparticles Featuring Polyzwitterionic Coatings. Polymers.

[10] Ishihara K. (2019). Blood-Compatible Surfaces with Phosphorylcholine-Based Polymers for Cardiovascular Medical Devices. Langmuir.

[11] Baggerman J., Smulders M.M.J., Zuilhof H. (2019). Romantic Surfaces: A Systematic Overview of Stable, Biospecific, and Antifouling Zwitterionic Surfaces. Langmuir.

[12] Laschewsky A., Rosenhahn A. (2019). Molecular Design of Zwitterionic Polymer Interfaces: Searching for the Difference. Langmuir.

[13] Venault A., Chang Y. (2019). Designs of Zwitterionic Interfaces and Membranes. Langmuir.

[14] Carr L.R., Zhou Y., Krause J.E., Xue H., Jiang S. (2011). Uniform zwitterionic polymer hydrogels with a nonfouling and functionalizable crosslinker using photopolymerization. Biomaterials.

[15] Yang R., Jang H., Stocker R., Gleason K.K. (2014). Synergistic Prevention of Biofouling in Seawater Desalination by Zwitterionic Surfaces and Low-Level Chlorination. Adv. Mater..

[16] Le N.L., Ulbricht M., Nunes S.P. (2017). How Do Polyethylene Glycol and Poly(sulfobetaine) Hydrogel Layers on Ultrafiltration Membranes Minimize Fouling and Stay Stable in Cleaning Chemicals?. Ind. Eng. Chem. Res..

[17] Nakaya T., Li Y.J. (1999). Phospholipid polymers. Prog. Polym. Sci..

[18] Mueller A., O’Brien D.F. (2002). Supramolecular Materials via Polymerization of Mesophases of Hydrated Amphiphiles. Chem. Rev..

[19] Monge S., Canniccioni B., Graillot A., Robin J.-J. (2011). Phosphorus-Containing Polymers: A Great Opportunity for the Biomedical Field. Biomacromolecules.

[20] Hu G., Emrick T. (2016). Functional Choline Phosphate Polymers. J. Am. Chem. Soc..

[21] Wielema T.A., Engberts J.B.F.N. (1990). Zwitterionic polymers—II. Synthesis of a novel series of poly(vinylbetaines) and the effect of the polymeric structure on the solubility behaviour in water. Eur. Polym. J..

[22] Bonte N., Laschewsky A. (1996). Zwitterionic polymers with carbobetaine moieties. Polymer.

[23] Kathmann E.E., White L.A., McCormick C.L. (1997). Water soluble polymers: 70. Effects of methylene versus propylene spacers in the pH and electrolyte responsiveness of zwitterionic copolymers incorporating carboxybetaine monomers. Polymer.

[24] Favresse P., Laschewsky A. (2001). Synthesis and Investigation of New Amphiphilic Poly(carbobetaine)s Made from Diallylammonium Monomers. Polymer.

[25] Avci D., Lemopulo K., Mathias L.J. (2001). Cyclocopolymerization of allyl-acrylate quaternary ammonium salts with diallyldimethylammonium chloride. J. Polym. Sci. Part A Polym. Chem..

[26] Cao Z., Mi L., Mendiola J., Ella-Menye J.-R., Zhang L., Xue H., Jiang S. (2012). Reversibly Switching the Function of a Surface between Attacking and Defending against Bacteria. Angew. Chem. Int. Ed..

[27] Colak S., Tew G.N. (2012). Amphiphilic Polybetaines: The Effect of Side-Chain Hydrophobicity on Protein Adsorption. Biomacromolecules.

[28] Rössel C., Billing M., Görls H., Festag G., Grube M., Bellstedt P., Nischang I., Schacher F.H. (2017). Synthesis and modification of poly(ethyl 2-(imidazol-1-yl)acrylate) (PEImA). Polymer.

[29] Kurowska M., Eickenscheidt A., Al-Ahmad A., Lienkamp K. (2018). Simultaneously Antimicrobial, Protein-Repellent, and Cell-Compatible Polyzwitterion Networks: More Insight on Bioactivity and Physical Properties. ACS Appl. Bio Mater..

[30] Monroy Soto V.M., Galin J.C. (1984). Poly(sulphopropylbetaines): 1. Synthesis and characterization. Polymer.

[31] Laschewsky A., Zerbe I. (1991). Polymerizable and polymeric zwitterionic surfactants: 2. Surface activity and aggregation behaviour in aqueous systems. Polymer.

[32] Anton P., Laschewsky A. (1993). Zwitterionic polysoaps with reduced density of surfactant side groups. Makromol. Chem..

[33] Köberle P., Laschewsky A. (1994). Hydrophobically modified zwitterionic polymers: Synthesis, bulk properties, and miscibility with inorganic salts. Macromolecules.

[34] Köberle P., Laschewsky A., Lomax T.D. (1991). Interactions of a zwitterionic polysoap and its cationic analog with inorganic salts. Makromol. Chem. Rapid Commun..

[35] Willcock H., Lu A., Hansell C.F., Chapman E., Collins I.R., O’Reilly R.K. (2014). One-pot synthesis of responsive sulfobetaine nanoparticles by RAFT polymerisation: The effect of branching on the UCST cloud point. Polym. Chem..

[36] Woodfield P.A., Zhu Y., Pei Y., Roth P.J. (2014). Hydrophobically Modified Sulfobetaine Copolymers with Tunable Aqueous UCST through Postpolymerization Modification of Poly(pentafluorophenyl acrylate). Macromolecules.

[37] Chang C.-C., Letteri R., Hayward R.C., Emrick T. (2015). Functional Sulfobetaine Polymers: Synthesis and Salt-Responsive Stabilization of Oil-in-Water Droplets. Macromolecules.

[38] Hildebrand V., Laschewsky A., Päch M., Müller-Buschbaum P., Papadakis C.M. (2017). Effect of the Zwitterion Structure on the Thermo-responsive Behaviour of Poly(Sulfobetaine Methacrylate)s. Polym. Chem..

[39] Ghoussoub Y.E., Fares H.M., Delgado J.D., Keller L.R., Schlenoff J.B. (2018). Antifouling Ion-Exchange Resins. ACS Appl. Mater. Interfaces.

[40] Armitage B.A., Bennett D.E., Lamparski H.G., O’Brien D.F. (1996). Polymerization and Domain Formation in Lipid Assemblies. Adv. Polym. Sci..

[41] Puri A., Blumenthal R. (2011). Polymeric Lipid Assemblies as Novel Theranostic Tools. Acc. Chem. Res..

[42] Ishihara K., Mu M., Konno T., Inoue Y., Fukazawa K. (2017). The unique hydration state of poly(2-methacryloyloxyethyl phosphorylcholine). J. Biomater. Sci. Polym. Ed..

[43] Vasantha V.A., Jana S., Parthiban A., Vancso J.G. (2014). Halophilic polysulfabetaines - synthesis and study of gelation and thermoresponsive behavior. RSC Adv..

[44] Wen J., Weinhart M., Lai B., Kizhakkedathu J., Brooks D.E. (2016). Reversible hemostatic properties of sulfabetaine/quaternary ammonium modified hyperbranched polyglycerol. Biomaterials.

[45] Gal Y.-S., Jin S.-H., Park J.W., Lim K.T. (2013). Conjugated organic polymer from the uncatalyzed polymerization of 2-ethynylpyridine via the ring-opening of 1,3-propandiol cyclic sulfate. Synth. Met..

[46] Shao Q., Jiang S. (2014). Influence of Charged Groups on the Properties of Zwitterionic Moieties: A Molecular Simulation Study. J. Phys. Chem. B.

[47] Vasantha V.A., Jana S., Lee S.S.-C., Lim C.-S., Teo S.L.-M., Parthiban A., Vancso J.G. (2015). Dual hydrophilic and salt responsive schizophrenic block copolymers—Synthesis and study of self-assembly behavior. Polym. Chem..

[48] Vasantha V.A., Zainul Rahim S.Z., Jayaraman S., Junyuan G.H., Puniredd S.R., Ramakrishna S., Teo S.L.-M., Parthiban A. (2016). Antibacterial, electrospun nanofibers of novel poly(sulfobetaine) and poly(sulfabetaine)s. J. Mater. Chem. B.

[49] Arjunan Vasantha V., Junhui C., Ying T.B., Parthiban A. (2015). Salt-Responsive Polysulfabetaines from Acrylate and Acrylamide Precursors: Robust Stabilization of Metal Nanoparticles in Hyposalinity and Hypersalinity. Langmuir.

[50] Schönemann E., Laschewsky A., Rosenhahn A. (2018). Exploring the Long-Term Hydrolytic Behavior of Zwitterionic Polymethacrylates and Polymethacrylamides. Polymers.

[51] Nizardo N., Schanzenbach D., Schönemann E., Laschewsky A. (2018). Exploring Poly(ethylene glycol)-Polyzwitterion Diblock Copolymers as Biocompatible Smart Macrosurfactants Featuring UCST-Phase Behavior in Normal Saline Solution. Polymers.

[52] Koc J., Schönemann E., Amuthalingam A., Clarke J., Finlay J.A., Clare A.S., Laschewsky A., Rosenhahn A. (2019). Low Fouling Thin Hydrogel Coatings Made of Photo-crosslinked Polyzwitterions. Langmuir.

[53] Buller J., Laschewsky A., Wischerhoff E. (2013). Photoreactive Oligoethylene Glycol Polymers—Versatile Compounds for Surface Modification by Thin Hydrogel Films. Soft Matter.

[54] Terayama Y., Kikuchi M., Kobayashi M., Takahara A. (2011). Well-Defined Poly(sulfobetaine) Brushes Prepared by Surface-Initiated ATRP Using a Fluoroalcohol and Ionic Liquids as the Solvents. Macromolecules.

[55] Galin M., Marchal E., Mathis A., Meurer B., Monroy Soto Y.M., Galin J.C. (1987). Poly(sulphopropylbetaines): 3. Bulk properties. Polymer.

[56] Lowe A.B., Billingham N.C., Armes S.P. (1999). Synthesis and Properties of Low-Polydispersity Poly(sulfopropylbetaine)s and Their Block Copolymers. Macromolecules.

[57] Hildebrand V., Laschewsky A., Zehm D. (2014). On the hydrophilicity of polyzwitterion poly (N,N-dimethyl-N-(3-(methacrylamido)propyl)ammoniopropane sulfonate) in water, deuterated water, and aqueous salt solutions. J. Biomater. Sci. Polym. Ed..

[58] Salamone J.C., Volksen W., Olson A.P., Israel S.C. (1978). Aqueous solution properties of a poly(vinyl imidazolium sulphobetaine). Polymer.

[59] Monroy Soto V.M., Galin J.C. (1984). Poly(sulphopropylbetaines): 2. Dilute solution properties. Polymer.

[60] Wielema T.A., Engberts J.B.F.N. (1990). Zwitterionic polymers—III. Salt effects on the solubility of poly(vinyl sulphobetaines) and poly(vinyl betaines) in aqueous solution. Eur. Polym. J..

[61] Kathmann E.E.L., White L.A., McCormick C.L. (1997). Water-Soluble Polymers. 73. Electrolyte- and pH-Responsive Zwitterionic Copolymers of 4-[(2-Acrylamido-2-methylpropyl)- dimethylammonio]butanoate with 3-[(2-Acrylamido-2-methyl- propyl)dimethylammonio]propanesulfonate. Macromolecules.

[62] Delgado J.D., Schlenoff J.B. (2017). Static and Dynamic Solution Behavior of a Polyzwitterion Using a Hofmeister Salt Series. Macromolecules.

[63] Wang T., Kou R., Liu H., Liu L., Zhang G., Liu G. (2016). Anion Specificity of Polyzwitterionic Brushes with Different Carbon Spacer Lengths and Its Application for Controlling Protein Adsorption. Langmuir.

[64] Shao Q., Jiang S. (2015). Molecular Understanding and Design of Zwitterionic Materials. Adv. Mater..

[65] Prucker O., Naumann C.A., Rühe J., Knoll W., Frank C.W. (1999). Photochemical Attachment of Polymer Films to Solid Surfaces via Monolayers of Benzophenone Derivatives. J. Am. Chem. Soc..

[66] Beines P.W., Klosterkamp I., Menges B., Jonas U., Knoll W. (2007). Responsive Thin Hydrogel Layers from Photo-Cross-Linkable Poly(N-isopropylacrylamide) Terpolymers. Langmuir.

[67] Lin X., Fukazawa K., Ishihara K. (2015). Photoreactive Polymers Bearing a Zwitterionic Phosphorylcholine Group for Surface Modification of Biomaterials. ACS Appl. Mater. Interfaces.

[68] Hartleb W., Saar J.S., Zou P., Lienkamp K. (2016). Just Antimicrobial is not Enough: Toward Bifunctional Polymer Surfaces with Dual Antimicrobial and Protein-Repellent Functionality. Macromol. Chem. Phys..

[69] Martinez J.S., Kelly K.D., Ghoussoub Y.E., Delgado J.D., Keller T.C.S., Schlenoff J.B. (2016). Cell resistant zwitterionic polyelectrolyte coating promotes bacterial attachment: An adhesion contradiction. Biomater. Sci..

[70] Liu Q., Locklin J. (2018). Transparent Grafted Zwitterionic Copolymer Coatings That Exhibit Both Antifogging and Self-Cleaning Properties. ACS Omega.

[71] Prucker O., Brandstetter T., Rühe J. (2018). Surface-attached hydrogel coatings via C,H-insertion crosslinking for biomedical and bioanalytical applications (Review). Biointerphases.

[72] Laschewsky A., Rekai E.D., Wischerhoff E. (2001). Tailoring of Stimuli-Responsive Water-Soluble Acrylamide and Methacrylamide Polymers. Macromol. Chem. Phys..

[73] Kim M., Ondrusek B.A., Lee C., Douglas W.G., Chung H. (2018). Synthesis of lightly crosslinked zwitterionic polymer-based bioinspired adhesives for intestinal tissue sealing. J. Polym. Sci. Part A Polym. Chem..

[74] Chou Y.-N., Sun F., Hung H.-C., Jain P., Sinclair A., Zhang P., Bai T., Chang Y., Wen T.-C., Yu Q. (2016). Ultra-low fouling and high antibody loading zwitterionic hydrogel coatings for sensing and detection in complex media. Acta Biomater..

[75] Yang F., Liu Y., Zhang Y., Ren B., Xu J., Zheng J. (2017). Synthesis and Characterization of Ultralow Fouling Poly(N-acryloyl-glycinamide) Brushes. Langmuir.

[76] Yang W., Chen S., Cheng G., Vaisocherová H., Xue H., Li W., Zhang J., Jiang S. (2008). Film Thickness Dependence of Protein Adsorption from Blood Serum and Plasma onto Poly(sulfobetaine)-Grafted Surfaces. Langmuir.

[77] Yang W., Xue H., Li W., Zhang J., Jiang S. (2009). Pursuing “Zero” Protein Adsorption of Poly(carboxybetaine) from Undiluted Blood Serum and Plasma. Langmuir.

[78] Zhao C., Li L., Wang Q., Yu Q., Zheng J. (2011). Effect of Film Thickness on the Antifouling Performance of Poly(hydroxy-functional methacrylates) Grafted Surfaces. Langmuir.

[79] Liu Q., Singh A., Liu L. (2013). Amino Acid-Based Zwitterionic Poly(serine methacrylate) as an Antifouling Material. Biomacromolecules.

[80] Inoue Y., Onodera Y., Ishihara K. (2016). Preparation of a thick polymer brush layer composed of poly(2-methacryloyloxyethyl phosphorylcholine) by surface-initiated atom transfer radical polymerization and analysis of protein adsorption resistance. Colloids Surf. B.

[81] Utrata-Wesołek A., Wałach W., Anioł J., Sieroń A.L., Dworak A. (2016). Multiple and terminal grafting of linear polyglycidol for surfaces of reduced protein adsorption. Polymer.

[82] Yuan L., Qu B., Li J., Lv H., Yang X. (2019). Photoreactive benzophenone as anchor of modifier to construct durable anti-platelets polymer surface. Eur. Polym. J..

[83] Vogler E.A. (1998). Structure and reactivity of water at biomaterial surfaces. Adv. Colloid Interface Sci..

[84] Herrwerth S., Eck W., Reinhardt S., Grunze M. (2003). Factors that Determine the Protein Resistance of Oligoether Self-Assembled Monolayers—Internal Hydrophilicity, Terminal Hydrophilicity, and Lateral Packing Density. J. Am. Chem. Soc..

[85] Rosenhahn A., Schilp S., Kreuzer H.J., Grunze M. (2010). The role of “inert” surface chemistry in marine biofouling prevention. Phys. Chem. Chem. Phys..

[86] Kim J., Somorjai G.A. (2003). Molecular Packing of Lysozyme, Fibrinogen, and Bovine Serum Albumin on Hydrophilic and Hydrophobic Surfaces Studied by Infrared−Visible Sum Frequency Generation and Fluorescence Microscopy. J. Am. Chem. Soc..

[87] Bratek-Skicki A., Eloy P., Morga M., Dupont-Gillain C. (2018). Reversible Protein Adsorption on Mixed PEO/PAA Polymer Brushes: Role of Ionic Strength and PEO Content. Langmuir.

